# An Improved Model of Moderate Sleep Apnoea for Investigating Its Effect as a Comorbidity on Neurodegenerative Disease

**DOI:** 10.3389/fnagi.2022.861344

**Published:** 2022-06-29

**Authors:** Reno Roberts, Mark J. Wall, Ingke Braren, Karendeep Dhillon, Amy Evans, Jack Dunne, Simbarashe Nyakupinda, Robert T. R. Huckstepp

**Affiliations:** ^1^School of Life Sciences, University of Warwick, Coventry, United Kingdom; ^2^University Medical Center Eppendorf, Vector Facility, Institute for Experimental Pharmacology and Toxikology, Hamburg, Germany

**Keywords:** cognitive decline, sleep apnoea (SA), neuroinflammation, neurodegeneration, sleep deprivation (SD)

## Abstract

Sleep apnoea is a highly prevalent disease that often goes undetected and is associated with poor clinical prognosis, especially as it exacerbates many different disease states. However, most animal models of sleep apnoea (e.g., intermittent hypoxia) have recently been dispelled as physiologically unrealistic and are often unduly severe. Owing to a lack of appropriate models, little is known about the causative link between sleep apnoea and its comorbidities. To overcome these problems, we have created a more realistic animal model of moderate sleep apnoea by reducing the excitability of the respiratory network. This has been achieved through controlled genetically mediated lesions of the preBötzinger complex (preBötC), the inspiratory oscillator. This novel model shows increases in sleep disordered breathing with alterations in breathing during wakefulness (decreased frequency and increased tidal volume) as observed clinically. The increase in dyspnoeic episodes leads to reduction in REM sleep, with all lost active sleep being spent in the awake state. The increase in hypoxic and hypercapnic insults induces both systemic and neural inflammation. Alterations in neurophysiology, an inhibition of hippocampal long-term potentiation (LTP), is reflected in deficits in both long- and short-term spatial memory. This improved model of moderate sleep apnoea may be the key to understanding why this disorder has such far-reaching and often fatal effects on end-organ function.

## Introduction

Current estimates of the prevalence of moderate to severe (≥15 events.h^−1^) sleep apnoea (SA, >10 s of respiratory depression, ~2 missed breaths during sleep) are ~4% for men and ~2% for women in the United Kingdom (Young et al., 1993). However, recent large cohort studies show that 91% of SA cases are undetected (Tan et al., 2016), with an occurrence of ~50% of men and ~25% of women over 40 (Heinzer et al., 2015).

During rapid eye movement (REM) sleep, individuals experience increased upper airway resistance (Rowley et al., 1998) due to tonic motor inhibition lowering muscle tone (Xie, 2012). This increases vulnerability to repetitive pharyngeal collapse and cessation in airflow even in the presence of respiratory effort, otherwise known as obstructive sleep apnoea (OSA) (Remmers et al., 1978). During non-REM (NREM) sleep, loss of descending inputs in respiratory centers means control of breathing is solely modulated by metabolic factors, and breathing is more reliant on chemoreception (Xie, 2012). Thus, any defects in the respiratory microcircuit will be exposed, leading to loss of drive in respiratory muscles, termed central sleep apnoea (CSA) (Xie, 2012). Whilst it is believed that OSA is primarily a REM-related disorder with CSA occurring in NREM, both CSA and OSA can occur during either NREM or REM sleep (Loadsman and Wilcox, 2000; Gupta et al., 2016). In all cases, patients experience intermittent hypoxic hypercapnia following periods of apnoea (>90% reduction in ventilation) and hypopnoea (>50% reduction in ventilation) (Veasey and Rosen, 2019).

SA-related blood/gas changes induce oxidative imbalance, facilitating the generation and buildup of reactive oxygen species (ROS), leading to the development of systemic inflammatory-related biomarkers associated with chronic inflammation (Maniaci et al., 2021). Molecular markers of systemic inflammation are increased in patients with SA (Mcnicholas, 2009), elevating the risk of diseases with an underlying inflammatory component, e.g., cardiovascular disease (CVD) (Gottlieb et al., 2010), diabetes (Reutrakul and Mokhlesi, 2017), and dementia (Bahia and Pereira, 2015). Systemically, this can cause heart failure (HF) (Matuska et al., 2013), with mortality directly related to SA severity (Lanfranchi et al., 1999) and SA treatment decreasing mortality (Javaheri et al., 2011) and morbidity (Lavergne et al., 2015). SA causes endothelial dysfunction through inflammation, which is capable of impairing cerebral blood flow (Zuliani et al., 2008). In addition, an increase in hypertension (Calbet, 2003) and atherosclerosis (Drager et al., 2011) in SA patients elicits recurrent strokes (Brown et al., 2019). Furthermore, the hypercapnic hypoxia leads directly to oxidative stress and neuroinflammation (Dusak et al., 2013; Yang et al., 2013). All of these brain insults, individually or in combination, decrease hippocampal and grey matter volume (Dusak et al., 2013; Yang et al., 2013), resulting in memory deficits and cognitive decline (Yang et al., 2013; Bahia and Pereira, 2015).

Current animal models of SA used to study these pathways and associations are severely flawed: *chronic intermittent hypoxia* replicates the hypoxia observed during SA, but increased compensatory breathing leads to hypocapnia, the *opposite* of the clinical condition. Airway occlusion replicates the blood gas changes that occur during apnoea, but the frequency and duration of apnoeas *do not* replicate the clinical presentation. In both of these models, the simulated “apnoeas” continued after arousal or occur during wakefulness, initiating the defense response, which is physiologically *unrealistic* and will contribute to any related comorbidities. A recent model of SA used substance P, a neuromodulator that binds to neurokinin-1 receptors (NK1Rs) on neurones in the respiratory rhythm generator, conjugated to saporin, a neurotoxin (SP-SAP) (Mckay and Feldman, 2008). Emulating this model, using a genetic approach via targeted viral transduction, we have developed an improved model of moderate SA that only displays apnoeas during natural sleeping patterns. Furthermore, our model displays both systemic and neural inflammation, inducing altered neuronal activity in the hippocampus and cognitive deficits. This novel model of moderate SA can be utilized for investigating the influence of SA on associated conditions such as neurodegenerative disease.

## Methods

### Recombinant Adeno-Associated Virus (*rAAV*) Vector Particle Production and Purification

rAAVs were produced in Sf9 insect cells. rAAV transfer plasmids were derived from pFBGR (kindly provided by Robert Kotin, National Heart, Lung and Blood Institute, National Institutes of Health, Bethesda, MD, United States). Diphtheria toxin fragment A (DTA) followed by IRES and GFP as a fluorescent marker was inserted into pFBGR under the control of a human synapsin promoter by InFusion cloning. Two fragments were generated by PCR using PrimeStar GLX Polymerase (Clontech laboratories, Mountain View, CA, United States): the human synapsin promoter was amplified from pAAV-Syn-Venus (Tang et al., 2009) using 5′-tctagaaatattaaggtacgggaggtacttgg and 5′- GATCcttgctagcagcttgaattcgactgcgctctca. DTA-IRES-GFP was amplified from pAAV-DTA-IRES-GFP (kindly donated by Prof. Alex Gourine, UCL) using 5′-ctgctagcaagGATCcaccATGGATCCTGATGATGTTGTTG and 5′-gggcgaattgggtaccTTACTTGTACAGCTCGTCCATGC. pFBGR was cut with MluI and KpnI, and both fragments were inserted simultaneously using the InFusion HD Cloning Kit (Clontech laboratories, Mountain View, CA, United States) according to the recommendations of the manufacturer to generate the AAV transfer plasmid pFBGR-Ultra Syn DTA-IRES-GFP. Final AAV transfer plasmids were confirmed by restriction digest, by PCR using the specific primers mentioned above, and by sequencing.

For packaging of AAV serotype 1 particles, pSR651 (Addgene plasmid 65213, addgene, Watertown, MA, United States) was employed. For packaging of AAV serotype 2 particles, pSR657 (Addgene plasmid 65214, addgene, Watertown, MA, United States) was employed (Smith et al., 2009). For producing rAAV serotype 9 particles, pFastBacDual (Life Technologies, CA, United States) was employed, and rep2 and cap9 were introduced (Chen, 2008).

To monitor Sf9 transduction efficiency during rAAV production, GFP was introduced into pFBGR under the control of a baculoviral late basic promoter (pB), and mCherry was introduced into pSR651 and pSR657, generating pFBGR-Ultra GFP and pSR651-Ultra mCherry and pSR657-Ultra mCherry (“Ultra-Bac,” Philipps et al., 2005). pB was amplified from Baculovirus Genomic DNA using 5′-GCAATTGTTGTTGTTAAATTCCGTTTTGCGACGATGC and GTTTAAATTGTGTAATTTATGTAGCTG. GFP was amplified from pscAAV-GFP, and mCherry was amplified from pSicoR-Ef1a-mCh-Puro-GFPi (Addgene plasmid 31848, addgene, Watertown, MA, United States) using 5′-TTACACAATTTAAACGctagcATGGTGAGCAAGGGCGAGG and 5′- tgcaataaacaagttaacTTACTTGTACAGCTCGTCCATGC.

Baculovirus genomic DNA carrying AAV genomic components and packaging elements was produced according to the Bac-to-Bac Expression System (Life Technologies, CA, United States) and according to the recommendations of the manufacturer. *E.coli* DH10Bac were transformed, and colonies were grown on kanamycin/tetracyclin/gentamycin plates supplemented with IPTG/Bluo-Gal. Large white colonies were propagated in 5 mL of 2YT medium containing kanamycin/tetracyclin/gentamycin overnight. Baculoviral DNA was extracted by Miniprep alkaline lysis, precipitated with 2-propanol, and resuspended in 50 μL of ddH_2_O. Clones were verified by PCR using Synapsin/T7 promoter specific primers.

rAAVs were produced according to “titreless infected-cell preservation and scale-up (TIPS)” protocol (Wasilko et al., 2009). Therefore, Sf9 cells were seeded on 12-well plates, and baculovirus DNAs derived from pFBGR-Ultra Syn DTA-IRES-GFP, pSR651-Ultra mCherry, pSR657-Ultra mCherry, and pFastBacDual-Ultra rep2 cap9 were transfected with the recombinant baculoviral genomic DNA using TransIT-Insect (MoBITec, Göttingen, Germany) according to the recommendations of the manufacturer. After 3 days, transfection could be monitored by mCherry or GFP fluorescence. Sf9 cells were collected, and 2E+07 fresh Sf9 cells were infected with this primary stock. After 1 day, vital fluorescing Sf9 cells were collected and frozen at 2E+6 cells BIIC stocks in InsectXpress/10% DMSO, also known as baculovirusinfected insect cells (BIIC).

For production of rAAV, ~4E+08 fresh Sf9 cells (Merck, NJ, United States) were cultivated in InsectXpress Medium (Lonza, Basel, Switzerland) at a density of 1–2E+6 cells·mL^−1^; ~2E+06 Sf9 BIIC stocks containing the expression cassette Syn DTA-IRES-GFP and ~2E+06 Sf9 BIIC stocks containing the desired AAV packaging elements were added. After 4 days, cells were harvested and spun down for 20 min at 2,000 × g. The cell pellet was resuspended in lysis buffer (50 mM Tris base, 100 mM NaCl, 5 mM MgCl_2_, pH 8.5), and viral particles were released from the nucleus by three freeze-thaw cycles. Any virus released into the supernatant was precipitated by incubation overnight in 10% PEG-8000/1 M NaCl at 4°C and then centrifuged for 30 min at 4°C and 3,000 × g. The resulting pellet was resuspended in lysis buffer and combined with cell lysate. Benzonase (Merck KGaA, Darmstadt, Germany; final concentration 50 U·mL^−1^) was added and incubated for 1 h at 37°C. Cell debris was pelleted for 20 min at 12,000 × g and vector containing lysates were purified using iodixanol step gradients. Iodixanol 40% layers were harvested, and iodixanol was removed by ultrafiltration (Amicon Ultra-4 Centrifugal Filter Unit, 50-kDa cutoff; Millipore, Burlington, MA, United States).

The genomic titres of DNAse-resistant recombinant AAV6 particles were determined by quantitative PCR using SYBR Green qPCR Master MIX 2 (Thermo Fisher Scientific, Waltham, MA, United States) and an ABI 7900 HT cycler (ABI, Waltham, MA, United States). Viral vectors were quantified using T7/SV40 specific primers (5′-cctatagtgagtcgtattacgcgc and 5′- gctgcaataaacaagttgggccat).

Real-time PCR was conducted at 10 μL with 0.3 μM for each primer. Fluorescence was measured at the end of each annealing phase. AAV transfer plasmids were employed as a copy number standard. A standard curve for quantification was generated by serial dilutions of the respective vector plasmid DNA. The cycling conditions were as follows: 50°C for 2 min, 95°C for 10 min, followed by 35 cycles of 95°C for 15 s and 60°C for 60 s. Calculations were performed using the SDS 2.4 software (ABI, Waltham, MA, United States).

### Virus Handling

AAV2-9: syn-DTA-GFP (AAV:syn-DTA) at a titer of 3.87 × 10^13^ VP·mL^−1^ was aliquoted and stored at −80°C. On the day of injection, viruses were removed and held at 4°C, loaded into graduated glass pipettes (Drummond Scientific Company, Broomall, PA, United States), which were placed into a pippette holder for pressure injection. The AAV:syn-DTA used the synapsin promoter, transducing neurones with higher tropism for the AAV2-9 subtype; it did not transduce non-neuronal cells.

### Viral Transduction of preBötC Neurones

**Figure 1:**
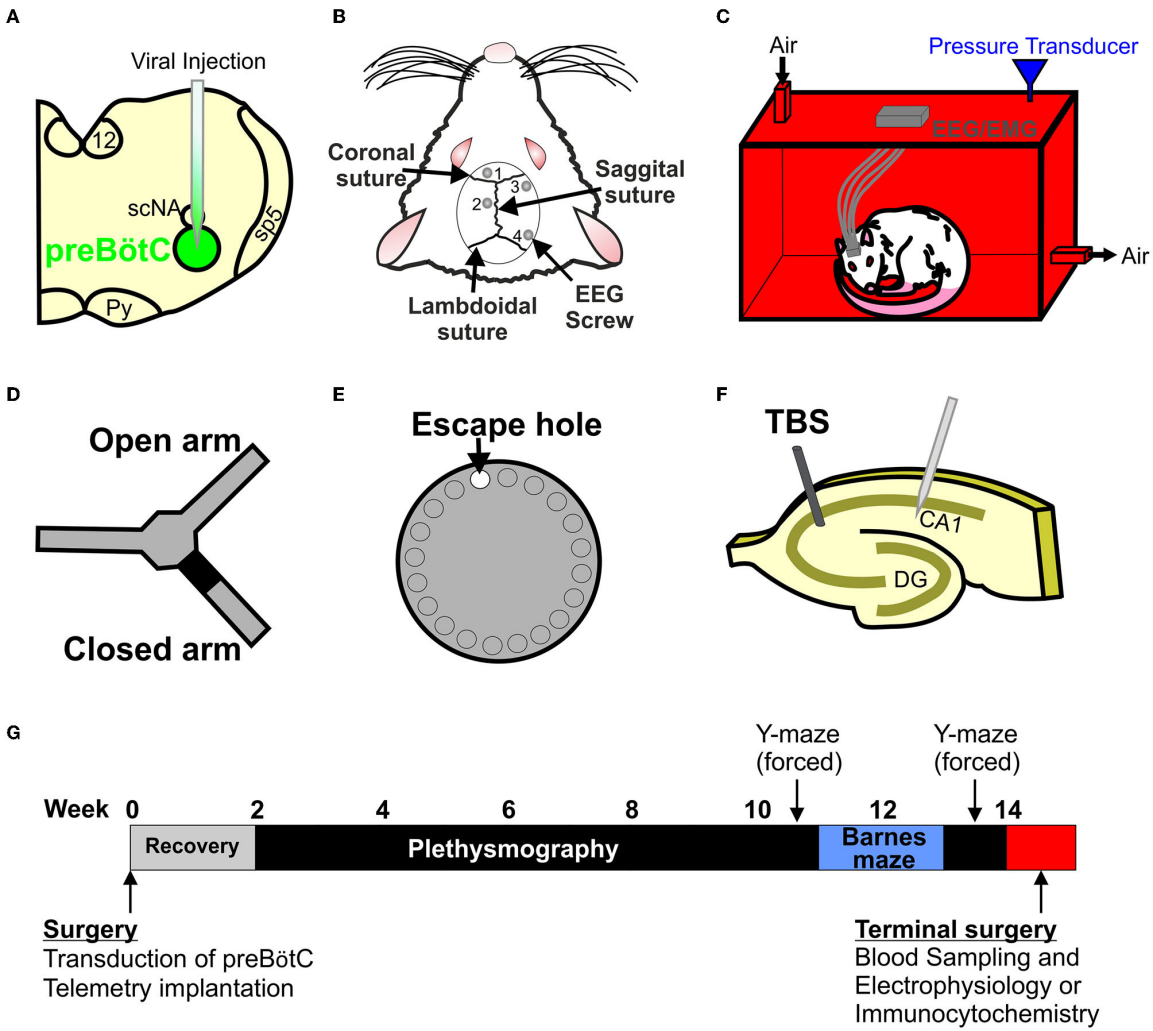
Creating and testing a new model of sleep apnoea (SA). **(A)** Stereotaxic injection of AAV2-9: syn-DTA-GFP into the preBötzinger complex (preBötC) was performed to create a stable lesion in the inspiratory oscillator. **(B)** EEG and EMG electrodes were used to determine sleep-wake states. **(C)** Rats were placed into a custom built 4.5 L plethysmographic chamber to record breathing and EEG and EMG signals to identify SA. **(D)** Y-maze (forced alteration) was used to determine short-term memory. **(E)** Barnes maze was used to test long-term memory. **(F)** Long-term potentiation (LTP) recordings were made to test for neurophysiological changes in the hippocampus. **(G)** Timeline of the experimental paradigm.

Adult male Sprague Dawley rats, between 6–8 months of age, were pseudorandomized by order and surgery type. Sham-operated rats weighed 437 ± 82g (*n* = 17), whilst DTA-injected rats weighed 456 ± 61g (*n* = 11). Three animals were excluded because of cortical brain injury caused by placement of EEG electrodes. Rats were anesthetized via intramuscular injection with ketamine (100 mg·kg^−1^; Covetrus, Dumfries, United Kingdom) and medetomidine (250 μg·kg^−1^; Covetrus, Dumfries, United Kingdom). Adequate anesthesia was maintained with 0.5–2% isoflurane (Piramal Healthcare, Mumbai, India) in pure oxygen (1 L·min^−1^) throughout the surgery as required. Rats received a presurgical subcutaneous injection of atropine (120 μg·kg^−1^; Westward Pharmaceutical Co., Eatontown, NJ, United States) and meloxicam (2 mg·kg^−1^; Norbrook Inc., Lenexa, KS, United States). Rats were placed prone into a digital stereotaxic apparatus (Kopf Instruments, Tujunga, CA, United States) on a heating pad (TCAT 2-LV: Physitemp, Clifton, NJ, United States), and body temperature was maintained at a minimum of 33°C via a thermocouple; mild hypothermia during surgery reduced blood loss and significantly improved post-surgical recovery. The head was angled so the nose bar was −18 mm below the intra-aural line. The injection arm was angled at 23°. Graduated glass pipettes containing the virus were placed stereotaxically into the preBötC (Figure 1A). The preBötC was defined as the area ventral to the semi-compact nucleus ambiguus (NA; coordinates: ±1.95 mm lateral, −0.2 mm rostral and +0.4 mm caudal, and −2.95 mm ventral from the obex; Figure 1A). The virus solution was pressure injected (~300 nL per site) bilaterally into all 4 sites to ensure good coverage of the preBötC. Pipettes were left in place for 3–5 min to prevent backflow of virus solution up the pipette track. Postoperatively, rats received subcutaneous injections of buprenorphine (100 μg·kg^−1^; Reckitt Benckiser, Slough, United Kingdom) and atipamezole (1 mg·kg^−1^; Vetoquinol, Northamptonshire, United Kingdom).

### Control Surgeries

Given the nature of the model, two separate controls were performed to account for different potential confounding factors.

Given 1 molecule of DTA is all that is required to induce apoptosis (Yamaizumi et al., 1978), any cell that expresses the plasmid undergoes apoptosis. Neurones of the preBötC of DTA-transduced rats are healthy and plasmid-free; we found no expression of GFP in transduced cells from injected rats (*n* = 4). For our sham surgeries, pipettes were lowered 2 mm dorsoventral from the obex, with the same mediolateral and rostral coordinates. These rats will have been exposed to all of the same surgical procedures, but without any damage to the preBötC; allowing us to compare preBötCs containing a reduced number of healthy cells following DTA induced cell death in transduced rats with intact preBötCs in sham operated animals.

Preliminary experiments were conducted to determine injection number and volume. Injection volumes that did not cause SA were used as a control group. Rats were injected with ~300 nL of AAV:syn-DTA either unilaterally in the aforementioned rostral and caudal coordinates or bilaterally in the aforementioned rostral coordinates. Rats that only received two injections of virus, be it unilaterally or bilaterally, did not display any signs of SA. Given these rats will have experienced cell death in the preBötC and will have released DTA into the extracellular milieu there, these rats act as control for the presence of apoptosis and the DTA subunit. As these rats showed no increase in AHI compared to sham-operated rats, data from the two controls were pooled. Therefore, we controlled for a change in preBötC size from wild-type levels, where all remaining neurones in both groups are free from viral transduction, and we also controlled for apoptosis and DTA production within the preBötC.

### EEG and EMG Placement

Following viral injections, the head was leveled so the nose bar was set to 0 at the intraural line. Burr holes were drilled at 4 locations: (1) immediately rostral to the coronal suture in a mediolateral position central to the frontal bone plate on the left of the sagittal suture to the most lateral extent of the bone; (2) in a rostrocaudal position centered between the coronal and lamboidal sutures in a central position on the left parietal bone plate between the sagittal suture and the most lateral extent of the bone; (3) immediately caudal to the coronal suture in a position on the right parietal bone plate centered between the sagittal suture and the most lateral extent of the bone; (4) immediately rostral to the lamboidal suture in a position on the right parietal bone plate centered between the sagittal suture and the most lateral extent of the bone (Figure 1B). EMGs were placed bilaterally into the trapezius muscles. EEG electrodes (M1.4 × 3 mm Phillips Pan Head Machine Screws (DIN 7985H)—Stainless Steel (A2), SIP—M1.4-3-A2; Accu, Huddersfield, United Kingdom) and EMG wires were connected to custom-built headmounts. Headmounts were adhered to the skull with superbond dental cement (Prestige Dental, Bedworth, United Kingdom), with additional support from Vertex Orthoplast cold-curing orthodontic acrylic resin (Prestige Dental, Bedworth, United Kingdom). Rats were allowed 2 weeks to recover from surgery and for viral expression, with food and water *ad libitum* at an ambient temperature of 22 ± 2°C. Following recovery, experimenters were blinded to the condition of the rats.

### Plethysmography

The order of the rats was pseudorandomized each week. Rats were acclimated for 30 min to the chamber in their home cage. On a separate occasion, rats were acclimated to the plethysmography room with a 30 min recording session under experimental conditions in postsurgical week 2. Following acclimation, rats were recorded for a single 3 h session per week with the recordings continuing for 7 weeks between post-surgical weeks 3 and 9. Recordings were conducted during the light phase of the 12 h light/dark cycle. For recordings, rats were placed into a custom-built 4.5 L plethysmography chamber (Figure 1C), with an airflow rate of 2 L·min^−1^ and an average temperature of 23 ± 0.9°C; thermoneutral for rats housed at an ambient temperature of 22 ± 2°C (Poole and Stephenson, 1977). Rats did not show any sign of thermoregulation, e.g., panting, shivering, or hyper-locomotion, and they displayed normal sleeping patterns. Pressure transducer signals were amplified and filtered using the NeuroLog system connected to a 1401 interface (Digitimer, Welwyn Garden City, United Kingdom). All data were acquired on a computer using Spike2 software (Cambridge Electronic Design, Cambridge, United Kingdom). For our analysis, respiratory disturbances were defined as a reduction in minute ventilation by >50% (hypopnoea) or >90% (apnoea) for ≥1.8 s. This duration was selected as:

1. In humans, sleep-disordered breathing is defined as perturbed breathing for ≥10 s, which is ~2 breaths. In our experiments, rat respiratory rate was 105 breaths·min^−1^, or 2 breaths every 1.2 s. To ensure that we did not overestimate the severity of our model, we use a duration equivalent to 3 breaths.
2. Adult rats exposed to obstructive apnoeas >5 s in duration, display extreme O_2_ desaturations in all events (Farré et al., 2007). Using the equation of the line from values for 10 and 15 s apnoeas from Farré et al. (2007), which predicts a resting saturation of 99%, we extrapolated the point at which apnoeas would reach an oxygen desaturation of 4% as ~1.875 s.

Apnoeas and hypopnoeas were scored by hand, and the length of each event recorded. Respiratory parameters were measured during quiet wakefulness at the beginning of the recording. Airflow measurements were used to calculate: tidal volume (V_T_), signal trough at the end of expiration subtracted from the peak signal during inspiration, converted to mL following calibration, and standardized to body weight; respiratory frequency (*f*) calculated as breaths per minute using peak of inspiration to peak of inspiration averaged over 1 min. Minute ventilation (V_E_) was calculated as V_T_ × *f*.

### Sleep-Wake Recordings

Theta rhythms were obtained by differential recordings from electrodes 1 and 2 (see EEG and EMG Placement) positioned in the dorsal hippocampus and the frontal cortex, respectively (Figure 1B). Delta waves and neocortical EEG activity were obtained by differential recordings from electrodes 3 and 4 (Figure 1B). EEG signals were amplified 7,500-fold and bandpass-filtered at 5–70 Hz. EMG signals were recorded by differential recordings through the trapezoid wires (Figure 1B) and were amplified 4,500-fold and bandpass filtered at 50–1,500 Hz. Videos were recorded to aid with sleep-wake scoring. All data were acquired on a computer using Spike2 software. EEG and EMG signals were processed using the OSD4 script: EEGs were band-filtered with a power spectrum of 0–4 Hz (delta) and 6–10 Hz (theta), and smoothed with a 5 s time constant. EMGs were processed with a 5 s RMS time constant. The 3 h long recordings were separated in 5 s epochs. Sleep-wake scored epochs were categorized into 4 predefined classes: (1) WAKE: low-amplitude desynchronized EEG activity and high EMG power; (2) NREM: high-amplitude EEG delta waves and low EMG power; (3) REM: continuous low-amplitude EEG theta activity leading to high theta:delta (T:D) ratio, and no EMG power, muscular paralysis could be observed on the associated video; (4) DOUBT: epochs that could not be defined into the 3 previous categories. For consistency, AHI counts and duration are expressed as per hour of total sleep even when discussing specific sleep states. Sigh counts are expressed as per hour of sleep + quiet wakefulness.

### Barnes Maze

The Barnes maze is a 122-cm diameter circular maze with 20 holes: 19 false holes and one escape hole, all measuring 9 cm in diameter (Stoelting, Dublin, Ireland) (Figure 1D). The maze is 1 m off the ground. Spatial cues of different colours and shapes are spaced evenly around the maze at regular intervals in plain sight of the rats. The surface of the table is brightly lit (>1,500 lux) to create an adverse environment. The Barnes maze began on post-surgical week 10 with the order of the rats pseudorandomized before each session. Rats underwent a 12 day Barnes maze protocol: days 1–3 (learning phase): the escape hole (randomly assigned on day 1) contained an incentive (peanut butter); days 4–12 (acquisition phase): the incentive is provided in the home cage after the test, allowing the rats to be rewarded for completing the test but removing the olfactory stimulus from the maze. The task was completed when the rats' entire body entered the exit hole. For consistency between rats, behavioural and respiratory measurements were not taken on the same day, as plethysmography may affect Barnes maze performance. All runs were recorded using a camera system (Henelec Model 335 BWL; Sony, Surry, United Kingdom) attached to a computer for offline analysis (Any-MAZE v4.96; Stoelting, Dublin, Ireland). Total distance, duration, and exit errors were measured. Exit errors were counted as the number of times the rats visited the exit hole but remained on the maze. Heat maps of the rats' movements were created for determination of search strategy. These were: random, no consistent pattern with ≥ 2 crossings of the open field; serial, hole-by-hole progression with ≥ 3 consecutive holes visited; spatial, moving directly to within 2 holes of the exit hole and no deviation outside of the quadrant (Wall et al., 2018).

### Y Maze (Forced Alternation)

A spontaneous spatial novelty preference test was conducted using a radial arm maze (Stoelting, Dublin, Ireland), adapted into a Y-maze. Each arm was 50 cm long and 10 cm wide, with 13-cm-high walls (Figure 1E). The surface of the table was comfortably lit (150–250 lux), with lighting levels consistent across the maze. Spatial cues of different colours and shapes were placed at the end of each arm of the maze. The order of the rats was pseudorandomized before each session. All rats were placed into the same entry arm. For the first trial, one arm of the Y-maze was blocked according to a pseudorandom sequence; therefore, rats could either go down to the unblocked arm, entry arm, or central region. First trials lasted for 10 min. After 1 h, the rats underwent the second trial to assess short-term memory. The second trial was performed with access to all arms. The Y maze took place either before or after the Barnes maze (week 10 or week 13). The order of the Barnes maze and Y-maze was determined by pseudorandomization during the experimental planning phase. All runs were recorded using a camera system (Henelec Model 335 BWL; Sony Surry, United Kingdom) attached to a computer for offline analysis (Any-MAZE v4.96, Stoelting, Dublin, Ireland). Total distance, duration, and number of entries into each arm were measured. An entry into an arm was defined as placement of 2 paws into that arm.

### Immunology

Rats were anesthetized by inhalation of isoflurane (0.5–2%; Piramal Healthcare, Mumbai, India) in room air (1 L·min^−1^). Inferior vena cava blood samples were taken and placed on ice in a tube containing EDTA (1.5 mg·mL^−1^ final concentration in the sample). Samples were spun at 13,000 rpm at 4°C for 10 min, and plasma was collected. Samples were aliquoted and stored at −80°C. Samples underwent no more than 2 freezing cycles.

Plasma samples were chosen as the relative differences between cytokines in serum and plasma would not be expected to be different. However, cellular components such as platelets can synthesize and release cytokines (Chen et al., 2020), which contribute to the overall amount of cytokines in serum. Therefore, plasma is considered to be a better reflection of circulating or any humoral cytokines when measured by enzyme-linked immunosorbent assay (ELISA).

Plasma samples were run on rat inflammation calorimetric sandwich ELISA kits (EA-1201 Signosis, Santa Clara, CA, United States) testing 8 inflammatory cytokines (TNFα, IL-6, MCP-1, IFNγ, RANTES, MIP, IL-1a, and IL-1b) and rat noradrenaline whole antibody sandwich ELISA kits (CSB-E07022R Cusabio, Houston, TX, United States). Optical densities were converted to concentrations via standard curves using protein standards (EA-1202 Signosis, Santa Clara, CA, United States). As a consequence of the variability in ELISA kits, measurements that did not lie on the linear part of the curve were discarded, and samples were run again. To further reduce the impact of the variability of ELSIA kits, samples were pseudorandomized to kits and run in duplicate on different kits, with the exception of noradrenaline, which was measured using a single kit.

### Collection of Brain Tissue

Rats were humanely killed by overdose of inhalation of isoflurane and decapitated with a guillotine. Brains were rapidly removed. The medulla and one cortical hemisphere were placed in 4% paraformaldehyde (PFA) for immunocytochemistry. The other cortical hemisphere was placed in ice cold saline for electrophysiological studies.

### Immunocytochemistry

Brains were postfixed overnight in PFA (4°C) and cryoprotected in 30% sucrose. Brainstems and hippocampi were serially sectioned at 40 μm. Free-floating sections were placed in a blocking solution (phosphate buffered saline [PBS] + 5% bovine serum albumin + 0.1% Triton X-100) and incubated for 1 h. Slices were further incubated overnight in blocking solution and primary antibodies: Medulla, rabbit anti-substance *p* receptor—(1:500; ab5060, Merck Millipore, Watford, United Kingdom) and mouse anti-NeuN (1:100; MAB337, Merck Millipore, Watford, United Kingdom) antibody; Hippocampus, goat anti-Iba1 (1:83; ab5076, Abcam, Cambridge, United Kingdom) antibody. Slices were washed in blocking solution (6 × 5 min) and then incubated for 2–4 h in blocking solution containing secondary antibodies: Medulla, donkey anti-rabbit Alexa Fluor 568 (1:250; ab150074, Abcam, Cambridge, United Kingdom) or donkey anti-mouse Alexa Fluor 568 (1:250; A10037, Invitrogen, Waltham, MA, United States) antibody; Hippocampus, donkey anti-goat Alexa Fluor 488 (1:250; A11055, Invitrogen, Waltham, MA, United States). Slices were washed in PBS (6 × 5 min), mounted (Medulla: Cytoseal 60; Electron Microscopy Sciences, Hatfield, PA, United States. Hippocampus: Vectashield Antifade Mounting Medium with DAPI; Vectorlabs, Burlingame, CA, United States) and coverslipped.

The NK1R antibody has been used in 24 peer-reviewed publications and has been validated elsewhere. NK1R is present in the nucleus ambiguus, and staining of the compact and semi-compact ambiguus were observed acting as a positive control. NK1R staining was also absent from areas devoid of NK1R, such as the SP-5 tract (data not shown), providing a negative control. Given the pattern of staining, previous validation, and use of the antibody elsewhere, the NK1R antibody is specific for the NK1R. The NeuN antibody has been used in 1,085 peer-reviewed publications and has been extensively validated. NeuN staining appears to be nuclear, given size and shape, and was located in voids left by NK1R staining, which cannot penetrate the nucleus, acting as a positive control. NeuN staining was absent from areas devoid of neurones, such as the SP-5 tract (data not shown), providing a negative control. Given the pattern of staining, previous validation, and use of the antibody elsewhere, we are certain that the NeuN antibody is specific for the nuclei of neurones. The Iba1 antibody we selected has been used in 24 peer-reviewed publications and has been validated elsewhere. Cells expressing Iba1 staining have the distinctive shape of microglia, providing a positive control. Iba1 staining was largely absent from the wild-type tissue, which should be devoid of activated microglia, providing a negative control. Given the pattern of staining, previous validation, and use of the antibody elsewhere, we are certain that the Iba1 antibody is specific for activated microglia.

Slices were examined using a confocal fluorescent microscope (Zeiss 880; Zeiss, Jena, Germany) with Zen black and Zen blue software. For the medulla: to determine the efficacy of viral transduction, counts of NeuN positive cells were performed in representative 40-μm sections throughout transduced brainstems and within a 600-μm diameter circle below the semi-compact nucleus ambiguus (Mckay and Feldman, 2008). To establish loss of rhythm-generating preBötC neurones, following background subtraction, fluorescence intensity of NK1R staining was assessed in ImageJ within the same circle. For the hippocampus: to identify activated microglia, we counted DAPI-stained nuclei encapsulated with Iba1 anywhere in the visual field. This provided several advantages over the use of Iba1 alone. First, as we only stained every second slice, nuclei could only be present in a single slice, removing any possibility of overestimation by double counting. Iba1 staining around the nucleus is very distinct from the staining of the processes, allowing us to distinguish nuclei enveloped in Iba1 from nuclei that have Iba1-positive projections in close apposition to them. These differences are amplified by imaging a z-stack with a confocal microscope when compared to standard fluorescence microscopy.

### Extracellular Recording of *f*EPSPs in Hippocampal Slices

The order of the rats was pseudorandomized before LTP experiments began. 400-μm thick sagittal slices of the hippocampus and overlying cortex were prepared under standard conditions in ice-cold aCSF (124 mM NaCl, 3 mM KCl, 2 mM CaCl_2_, 26 mM NaHCO_3_, 1.25 mM NaH_2_PO_4_, 1 mM MgSO_4_, 10 mM D-glucose saturated with 95% O_2_-5% CO_2_, pH 7.5, containing an additional 10 mM MgCl_2_). Slices were transferred to an incubation chamber containing aCSF at 32 ± 1°C and incubated for 1 h. All tissue slices were made at 9 am in the morning to standardize the amount of time rats had to sleep prior to removal of tissue. Extracellular recordings of synaptic transmission and plasticity were carried out using two recording rigs to increase productivity. Data from the two rigs were not statistically different and so were pooled.

Recording on rig 1: Slices were transferred to the recording chamber submerged in aCSF (124 mM NaCl, 3 mM KCl, 2 mM CaCl_2_, 26 mM NaHCO_3_, 1.25 mM NaH_2_PO_4_, 1 mM MgSO_4_, and 10 mM D-glucose saturated with 95% O2–5% CO2; pH 7.5) and perfused at 4–6 mL·min^−1^ (32°C). Slices were placed on a grid allowing perfusion above and below the tissue, and all tubing (Tygon, Akron, OH, United States) was gas-tight (to prevent loss of oxygen). To record field excitatory postsynaptic potentials (*f* EPSPs), an aCSF-filled microelectrode was placed on the surface of the stratum radiatum in CA1 (Figure 1F). A bipolar concentric stimulating electrode (CBBPC75, FHC, Bowdoin, ME, United States) controlled by an isolated pulse stimulator model (2100, AM Systems, WA, United States) was used to evoke *f* EPSPs in the Schaffer collateral–commissural pathway (Figure 1F). Field *f* EPSPs were evoked every 30 s (0.03 Hz). Stimulus input/output (I/O) curves for *f* EPSPs were generated using 0.5 V steps between 1 and 5 V for slices (stimuli duration 0.2 ms). Paired-pulse ratio was measured over intervals ranging between 20 to 500 ms (ratio averaged for 5 repeats at each interval). For synaptic plasticity experiments, stimulus strength was set to produce an *f* EPSP slope ~40 % of the maximum response. Long-term potentiation (LTP) was induced by theta burst stimulation (TBS, 3 trains separated by 20 s, with a train consisting of 10 bursts of 4 pulses at 100 Hz, separated by 200 ms). Signals were filtered at 3 kHz and digitized online (10 kHz) with a Micro CED (Mark 2) interface controlled with Spike 2 software (Vs 6.1, Cambridge Electronic Design, Cambridge United Kingdom). The *f* EPSP slope was measured from a 1 ms linear region following the fiber volley. All I/O curves and PPF comparisons were made from this recording rig.

Recording on rig 2: *f* EPSPs were recorded as outlined for rig 1, except perfusion was set at 6–7 mL·min^−1^. A bipolar concentric stimulating electrode (CBBPC75, FHC, Bowdoin, ME, United States) was controlled by a DS3 isolated current stimulator (Digitimer, Welwyn Garden City, United Kingdom) with field *f* EPSPs evoked every 45 s. Stimulus input/output curves for *f* EPSPs were generated using 20-μA steps between 20 and 300 μA (stimulus duration 0.2 ms), stimulus strength was set to produce an *f* EPSP slope ~40 % of maximum response. Long-term potentiation was induced by theta burst stimulation (as above for rig 1). Signals were acquired using the WinLTP software (Vs 2.3; WinLTP Ltd., Bristol, United Kingdom).

### Experimental Design

For transduction, the order of rats, and experimental type (whether they received viral injections or were shams) were pseudorandomized. Experimenters were blinded to the condition of the rats. Rats were acclimated to the plethysmography chamber for 30 min during the 2^nd^ week post-surgery. Plethysmography began during the 3^rd^ week post-surgery and continued for 7 weeks. Rats then underwent 1 day of Y-maze, 12 days of Barnes maze, and the order of maze testing was pseudo randomized for each batch. Tissue collection and LTP experiments were performed one week after the final maze session. Experimenters were unblinded to the condition of the rats once analysis had been performed (Figure 1G).

Control rats that received only 2 injections were acclimated to the plethysmography chamber on the 2^nd^ week post-surgery. Plethysmography began on the 3^rd^ week post-surgery and continued for 6 weeks. Tissue collection was performed one week after the final plethysmography session.

### Data Analysis

All experimental units are an animal; all technical repeats are averaged to create a biological repeat for analysis. Outliers were removed after Iglewicz and Hoaglin's robust test for multiple outliers with an outlier criterion: modified Z score ≥ 3.5 (https://contchart.com/outliers.aspx) or Grubb's test (OriginLab, Northampton, MA, United States). Shapiro-Wilk tests were performed on sham-operated groups.

Data for preBötC cell counts (*p* = 0.5), NA cell counts (*p* = 0.8), BötC cell counts (*p* = 0.5), preBötC corona cell counts (*p* = 0.4), preBötC NK1R intensity (*p* = 0.08), AHI (*p* = 0.3), time spent in disordered breathing (*p* = 0.25), AHI by sleep state (DTA: *p* = 0.6; sham: *p* = 0.6), time spent in disordered breathing by sleep state (DTA: *p* = 0.5; sham: *p* = 0.5), V_T_ (*p* = 0.3), *f* (*p* = 0.1), V_E_ (*p* = 0.2), WAKE (*p* = 0.2), NREM (*p* = 0.1), REM (*p* = 0.4), Iba1 counts (*p* = 0.6), IFNγ (*p* = 0.06), IL-1a (*p* = 0.96), IL-1b (*p* = 0.4) and IL-6 (*p* = 0.3), CRP (*p* = 0.9), noradrenaline (*p* = 0.7), and Y-maze discrimination ratios (duration: *p* = 0.7; distance: *p* = 0.8) were deemed Gaussian by a Shapiro-Wilk test for normality and tested by an unpaired *t*-test. The data are expressed as mean ± SD.

Data for Barnes maze search strategies (*p* = 0.2) were deemed Gaussian by a Shapiro-Wilk test for normality and tested by a one-way repeated measures ANOVA with Bonferroni correction. The data are expressed as mean ± SD.

Data for Y-maze novel arm entries (*p* = 0.6), Y-maze novel duration (*p* = 0.98), Y-maze novel arm distance (*p* = 0.06), and paired pulse facilitation (*p* = 0.6) were deemed Gaussian by a Shapiro-Wilk test for normality and tested by a two-way repeated measures ANOVA with Bonferroni correction. The data are expressed as mean ± SD.

Data for the average duration of apnoeas during sleep state (NREM: *p* = 0.3) and sleep state (REM: *p* = 0.9), were deemed Gaussian by a Shapiro-Wilk test for normality and tested by a two-way ANOVA. The data are expressed as mean ± SD.

Data for CA1 Iba1 counts (*p* = 0.02), TNFα (*p* = 0.02), MCP-1 (*p* = 0.03), MIP (*p* = 0.01), RANTES (0.02), and Y-maze discrimination ratio for entries (*p* = 0.001) were deemed non-Gaussian by a Shapiro-Wilk test for normality and tested by a Kruskal-Wallis Test; 68% of the data are contained within 1 SD, and to make the data comparable, we calculated the data range from the 15^th^ to 85^th^ percentile, herein known as R_70_. Data are expressed as median followed by the R_70_ and displayed graphically as median with percentiles 15–85.

Data for Barnes duration (*p* = 0), Barnes maze distance (*p* = 0), Barnes maze exit errors (*p* = 0), and LTP (*p* = 0.0002) were deemed non-Gaussian by a Shapiro-Wilk test for normality and tested by a two-way repeated measures ANOVA with Sidak correction. The data are expressed as mean ± SD.

Data for input-output curves (*p* = 0.00005) were deemed non-Gaussian by a Shapiro-Wilk test for normality and tested by a two-way ANOVA with Sidak correction. The data are expressed as mean ± SD.

## Results

### AAV:Syn-DTA (DTA) Transduction of the preBötC Leads to a Lesion of Rhythm-Generating Neurones

**Figure 2:**
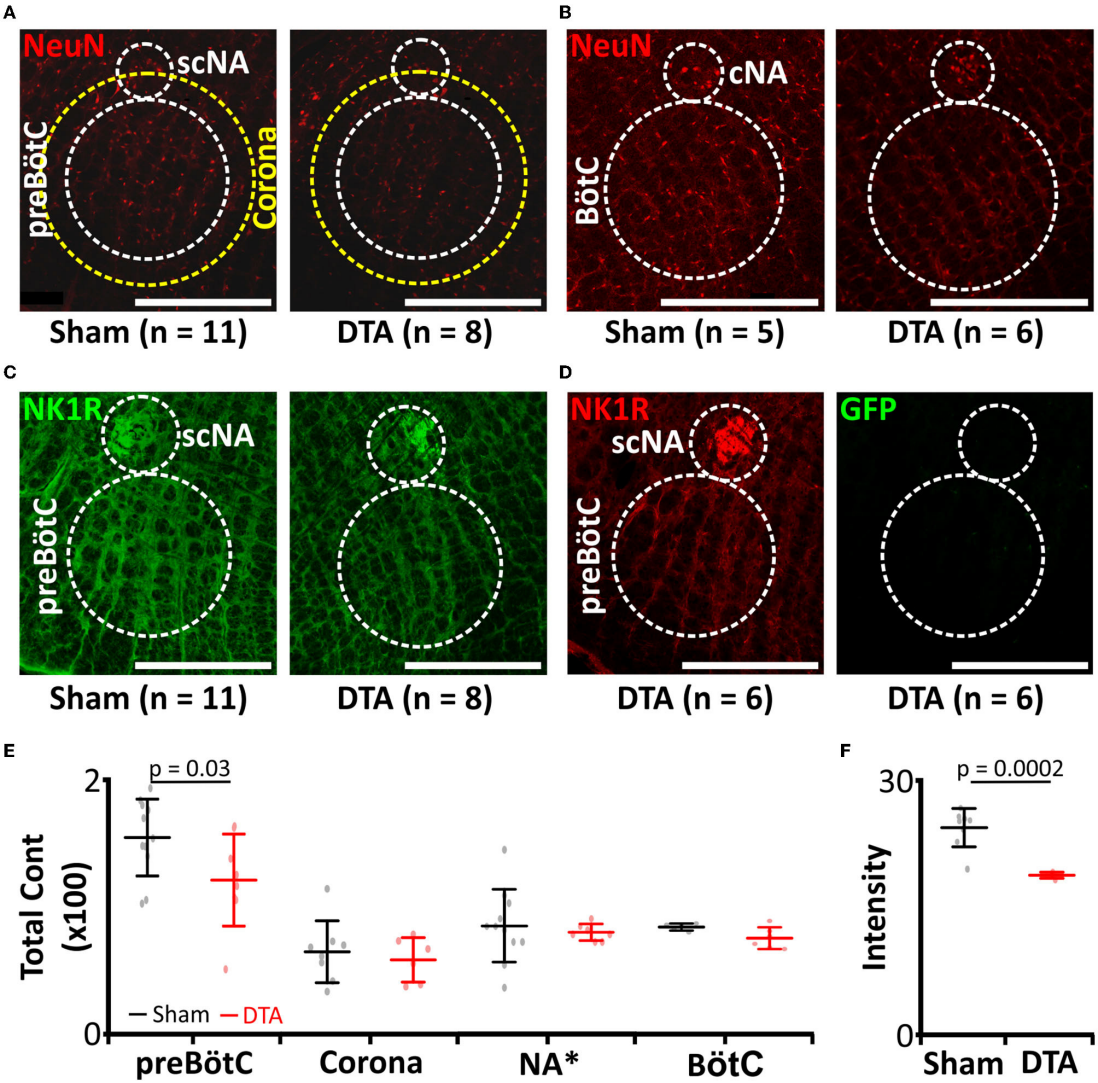
preBötC lesions create an experimental model of moderate SA. **(A–D)** Immunocytochemical staining following transduction of the preBötzinger complex (preBötC) with AAV2-9:Syn-DTA-GFP. DTA, diphtheria toxin subunit A; GFP, Green Fluorescent Protein. **(A)** Micrographs of neurones (NeuN: red) in the preBötC, scNA, and a 200 μm corona around the preBötC. scNA, semi-compact nucleus ambiguous. **(B)** Micrographs of neurones (NeuN: red) in the BötC. BötC, Bötzinger complex; cNA, compact nucleus ambiguous. **(C)** Micrographs of NK1R (green) in the preBötC and scNA. **(D)** Micrographs of GFP (green) in the preBötC and scNA. **(E)** Group data from neuronal counts from micrographs represented in **(A,B)**. *Scaling for NA is × 10. **(F)** Group data from NK1R intensities in the preBötC from micrographs represented in **(C)**.

Previously, substance-P conjugated to saporin (SP-SAP) was used to induce progressive loss of neurokinin 1 receptor-positive neurones (Mckay and Feldman, 2008). When SP-SAP is injected unilaterally into the inspiratory oscillator, the preBötzinger complex (preBötC), it induces SA once the size of lesion reaches ~25% of NK1R neurones (Mckay and Feldman, 2008). However, following apoptosis of the neurones, SP-SAP is released back into the extracellular milieu to be taken up by neighbouring neurones that express NK1R. Therefore, the lesion spreads beyond the confines of the preBötC and into the surrounding tissue including the NA [refer to Figure 5 in Mckay and Feldman (2008)]. We, therefore, took a different approach to create a stable unilateral partial lesion of the preBötC by stereotaxically targeted viral transduction of the preBötC with an adeno-associated virus (AAV), leading to expression of diphtheria toxin subunit-A (DTA; Figures 1A, 2A–F). Following apoptosis, DTA cannot enter neighbouring cells in the absence of the B subunit (Zalman and Wisnieski, 1984). This creates a stable lesion limited only to the transduced cells. This approach led to a 23% loss of neurones (sham: 1,695 ± 446 neurones, *n* = 11 vs. DTA: 1,240 ± 391 neurones, *n* = 8; *p* = 0.03; Figures 2A,E). This represented the loss of a significant portion of rhythm-generating cells (sham: 24.4 ± 2.3 NK1R intensity, *n* = 8 vs. DTA: 18.8 ± 0.4 NK1R intensity, *n* = 5; *p* = 0.0002; Figures 2C,F). DTA inhibits protein synthesis (Strauss and Hendee, 1959), and even a single molecule is enough to induce apoptosis (Yamaizumi et al., 1978). We found rats transduced with the DTA virus displayed no GFP expression (*n* = 4), even though preBötC neuronal numbers were reduced. Therefore, all neurones that expressed the virus underwent apoptosis. Viral injections were limited to the preBötC, as we saw no loss of neurones in: a 200-μm corona surrounding the preBötC (sham: 65 ± 24 average number of neurones per slice, *n* = 8 vs. DTA: 58 ± 17 average number of neurones per slice, *n* = 6; Figures 2A,E), the NA (sham: 13 ± 2 average number of neurones per slice, *n* = 11 vs. DTA: 12 ± 0 average number of neurones per slice, *n* = 7; Figures 2A,E), or the BötC (sham: 83 ± 3 average number of neurones per slice, *n* = 5 vs. DTA: 75 ± 9 average number of neurones per slice, *n* = 6; Figures 2B,E).

### DTA Lesions Lead to Sleep Disordered Breathing and Alterations in Respiration During Wakefulness

**Figure 3:**
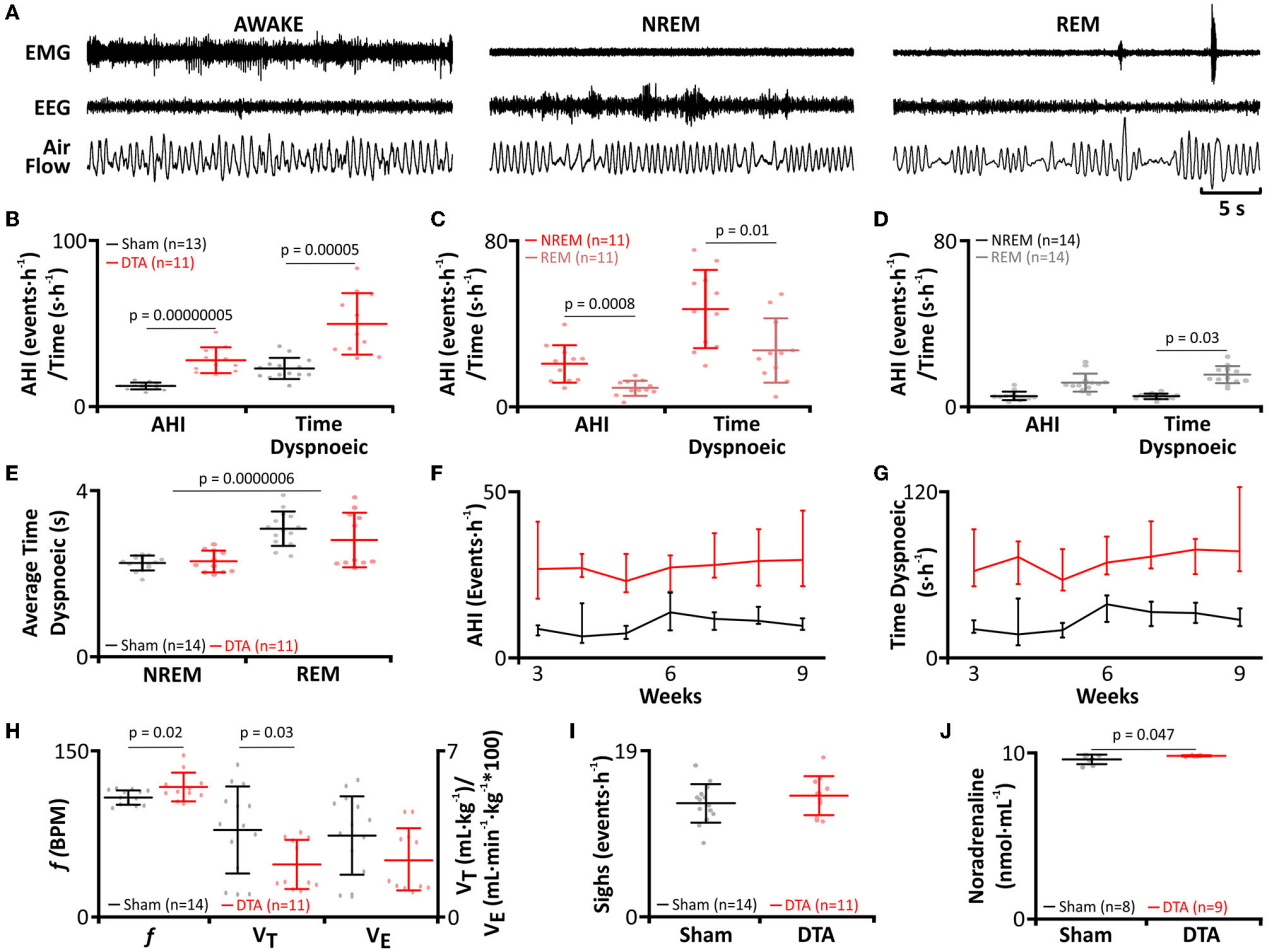
preBötC lesions create an experimental model of moderate sleep apnoea. **(A)** Plethysmograph traces from rats post-DTA injection, showing respiratory movements as measured by changes in air flow in a plethysmograph, with EEG and EMG electrodes for assigning sleep-wake state. **(B–J)** DTA transduced rats (red) compared to shams (black). **(B)** Frequency of breathing disturbances (AHI) and duration spent dyspnoiec (time). AHI, apnoea-hypopnoea index. **(C)** Frequency of breathing disturbances and duration spent dyspnoiec by sleep state, NREM (red) and REM (pink), in DTA transduced rats. **(D)** Frequency of breathing disturbances and duration spent dyspnoiec by sleep state, NREM (black) and REM (grey) in shams. **(E)** Average duration of a dyspnoea during NREM and REM. **(F)** Progression of AHI by week. **(G)** Progression of time spent dyspnoeic by week. **(H)** Respiratory parameters during wakefulness. Left axis shows values for frequency (*f*), right axis for tidal volume (V_T_) and minute ventilation (V_e_). **(I)** Frequency of sighing. **(J)** Plasma noradrenaline levels. Data represented as mean ± SD, with individual data points.

As viral transduction caused a significant lesion of the preBötC, we next studied how this affected breathing and whether this led to SA. Breathing of rats was measured by plethysmography (Figures 1C, 3A–J) and sleep-wake state via EEG/EMG recordings (Figures 1C, 3A). For assessment of SA severity, we measured the apnoea-hypopnoea index (AHI: sum of apnoeic + hypopnoeic events per hour of sleep), as is done clinically. A respiratory disturbance was defined as a reduction in minute ventilation of >1.8 s. The AHI was increased in DTA-injected rats (sham: 9 ± 3 incidences·hour of sleep^−1^, *n* = 14 vs. DTA: 30 ± 9 incidences·hour of sleep^−1^, *n* = 11; *p* = 0.00000005; Figure 3B), as was the total duration of dyspnoeic breathing (apnoeas + hypopnoeas) (sham: 26 ± 6 s, *n* = 14 vs. DTA: 74 ± 25 s, *n* = 11; *p* = 0.00005; Figure 3B). Disturbances in the DTA-transduced rats occurred during: 1) NREM sleep (21 ± 9 incidences·hour of sleep^−1^; 47 ± 7 s·hour of sleep^−1^; *n* = 11; Figure 3C) and REM sleep (9 ± 4 incidences·hour of sleep^−1^; 27 ± 6 s·hour of sleep^−1^; *n* = 11; Figure 3C). Therefore, NREM contributed a greater number (~70%) of breathing disturbances during sleep (*p* = 0.0008; Figure 3C), and a greater time spent dyspnoeic (*p* = 0.01; Figure 3C). The number of AHI events did not differ during sleep state in sham-operated rats (NREM: 5 ± 2 incidences·hour of sleep^−1^, *n* = 14 vs. REM: 5 ± 1 incidence·hour of sleep^−1^, *n* = 14; Figure 3D), although duration was increased in REM (NREM: 12 ± 4 s·hour of sleep^−1^, *n* = 14 vs. REM: 15 ± 4 s·hour of sleep^−1^, *n* = 14; *p* = 0.03; Figure 3D). The increase in time spent dyspnoeic in REM was due to an increase in duration of the average dyspnoeic event in this sleep state (NREM: 2.3 ± 0.2 s, *n* = 25 vs. REM: 2.9 ± 5 s, *n* = 25; *p* = 0.0000006; Figure 3E). Interestingly, the average duration of a dyspnoeic event was not affected by transduction with DTA (sham: 2.7 ± 0.5 s, *n* = 28 vs. DTA: 2.5 ± 5 s, *n* = 22; Figure 3E). The lesions created by DTA transduction created a stable phenotype with the number of incidences and time spent in dyspnoea remaining unchanged between weeks 3 and 9 post-surgery in terms of the number of incidences (*p* = 0.95) and duration spent in dyspnoea (*p* = 0.74) (Table 1; Figures 3F,G).

**Table 1:** Apnoea-hypopnoea index (AHI) per hour of sleep of diphtheria toxin fragment A (DTA)-transduced rats by week.

**Week**	**Count**	**Duration**
*3*	27 R_70_ 29	60 R_70_ 74
*4*	27 R_70_ 24	72 R_70_ 73
*5*	23 R_70_ 35	53 R_70_ 79
*6*	27 R_70_ 18	67 R_70_ 41
*7*	27 R_70_ 21	72 R_70_ 57
*8*	30 R_70_ 25	77 R_70_ 65
*9*	29 R_70_ 28	76 R_70_ 81

Rats with preBötC lesions had an increased *f* (sham: 95 ± 7 breaths·min^−1^, *n* = 14 vs. DTA: 105 ± 14 breaths·min^−1^, *n* = 11; *p* = 0.02; Figure 3H), with a reciprocal reduction in V_T_ (sham: 3.6 ± 2 mL·kg^−1^, *n* = 14 vs. DTA: 2.3 ± 1 mL·kg^−1^, *n* = 11; *p* = 0.03; Figure 3H). Although slight changes in frequency and tidal volume were seen, the overall integrity of the preBötC and, therefore, respiratory output was not diminished given that V_e_ remained unchanged (sham: 343 ± 163 mL·kg^−1^·min^−1^, *n* = 14 vs. DTA: 244 ± 129 mL·kg^−1^·min^−1^, *n* = 11; Figure 3H), as did sigh rate (Sham: 13 ± 9 incidences·h^−1^, *n* = 14 vs. DTA: 14 ± 7 incidences·h^−1^, *n* = 11; Figure 3I). Furthermore, we also found our model of sleep apnoea may have also affected the cardiovascular system given rats transduced with DTA displayed increased plasma noradrenaline (sham: 2 ± 0.3 ng·mL^−1^, *n* = 9 vs. DTA: 2.4 ± 0.4 ng·mL^−1^, *n* = 11; *p* = 0.047; Figure 3J), a marker of increased sympathetic output seen in patients with SA (Ziegler et al., 1997).

### Rats With Sleep Apnoea Have Reduced REM Sleep With no Effect on Time Spent in NREM

**Figure 4:**
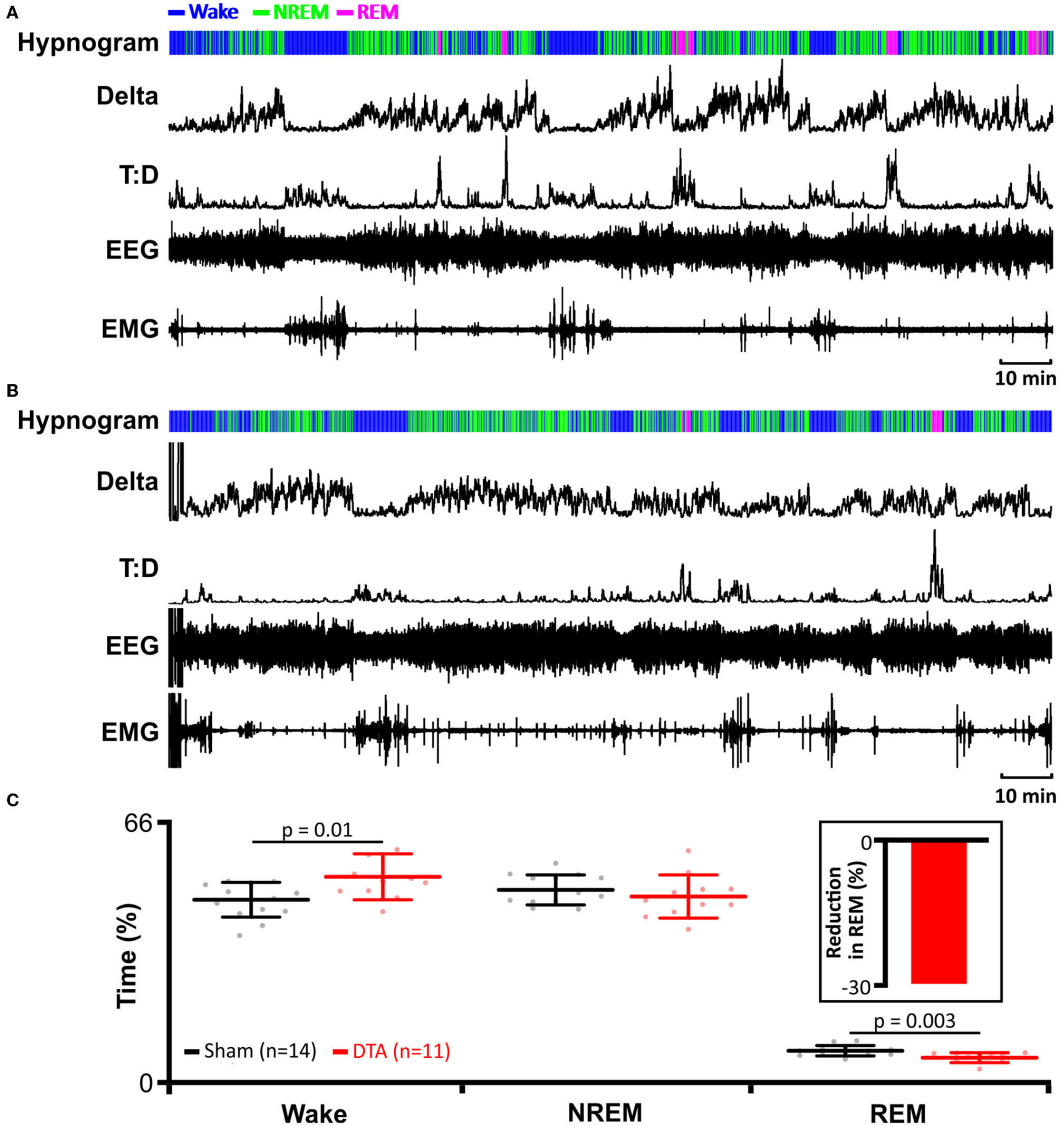
Induction of sleep apnoea leads to reduction in rapid eye movement (REM) sleep. **(A)** Data from an individual sham-operated rat. **(B)** Data from an individual DTA-injected rat. **(A,B)** EMG recordings were used to determine activity and to identify periods of REM and NREM sleep. EEG recordings were used to determine delta wave activity for NREM sleep and to calculate theta-delta ratio (T:D) for REM sleep. Hypnograms show time spent awake, and in REM and NREM sleep. **(C)** Time spent in wakefulness, and NREM and REM sleep in DTA transduced rats (red) compared to shams (black). DTA rats (red) spend less time in REM sleep (insert: percentage reduction) than shams (black), with lost REM time spent awake. Data are represented as mean ± SD, with individual data points.

Given the increase in dyspnoea observed during both phases of sleep, and that dyspnoeas can induce microarousals, we tested whether our DTA-injected rats exhibited any alteration in sleeping patterns. DTA lesions led to the onset of sleep disturbances (Figure 4). Apnoeas during NREM sleep were not associated with arousals (Figure 3A), and correspondingly, there was no effect on the percentage of time spent in NREM sleep (sham: 48 ± 4%, *n* = 14 vs. DTA: 46 ± 6 %, *n* = 11; Figures 4A–C). In the majority of cases, during REM sleep, arousals were associated with hyperpnoea that followed a series of dyspnoeas (Figure 3A), although in some cases arousal were coincident with the termination of an apnoea (not shown). The arousals caused by apnoeic events decreased the time rats spent in REM sleep by 30% (sham: 6 ± 1 breaths·min^−1^, *n* = 14 vs. DTA: 4 ± 1 breaths·min^−1^, *n* = 11; *p* = 0.003; Figures 4A–C), with all of the lost REM time being spent awake (sham: 46 ± 5%, *n* = 14 vs. DTA: 52 ± 6 breaths·min^−1^, *n* = 11; *p* = 0.01; Figures 4A–C). Whilst the effects of lesions of preBötC neurones on breathing phenotypes, AHI, and sleep architecture have been studied before (Mckay and Feldman, 2008), the physiological and neurological consequences of such lesions remain untested, something we wished to address.

### Rats With Sleep Apnoea Have Alterations in Systemic Inflammatory Markers and Plasma Catecholamine Levels

**Figure 5:**
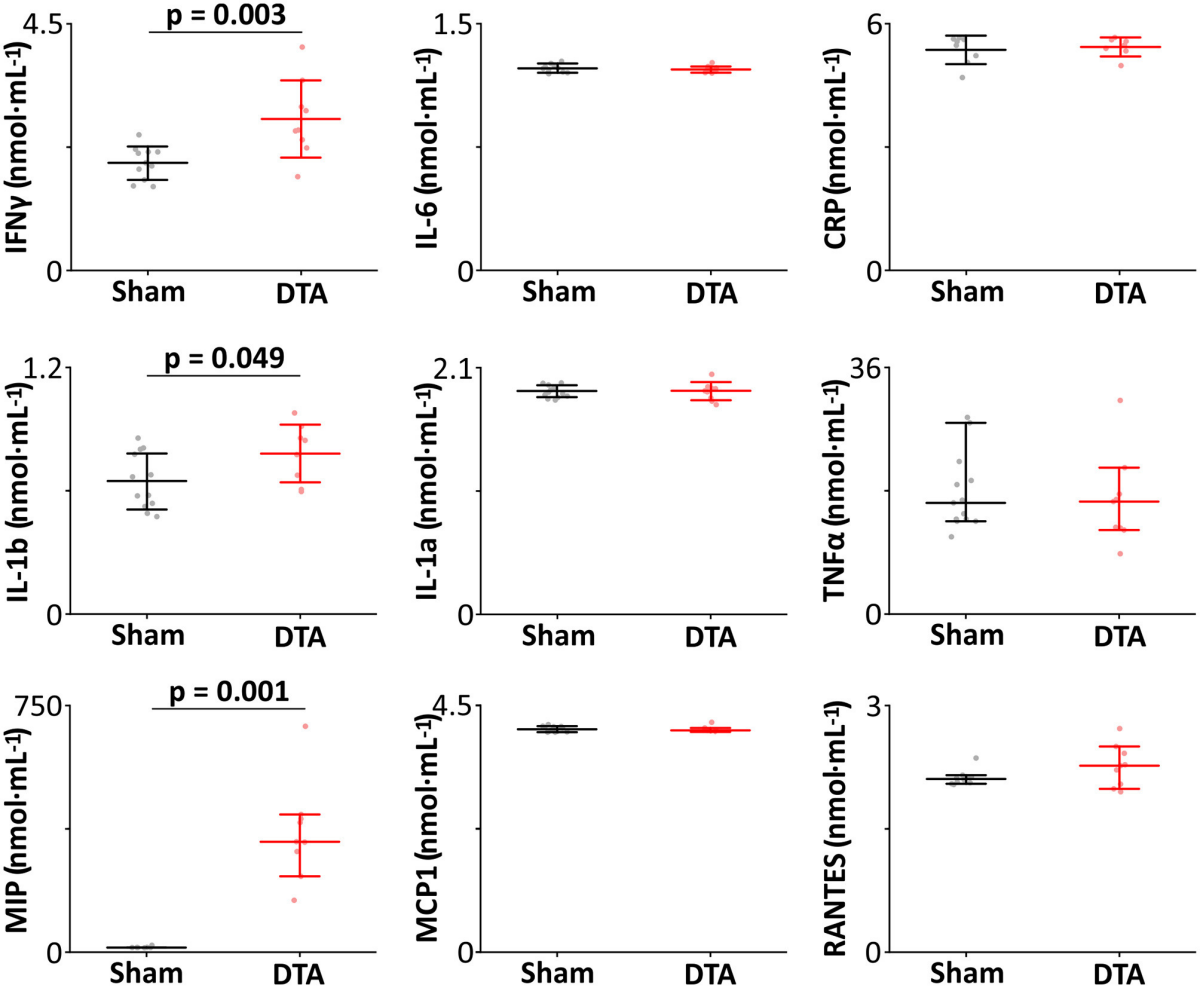
Induction of sleep apnoea leads to systemic inflammation. Inflammatory profiles (nmol·mL^−1^) from sham-operated (*n* = 14) and DTA-injected (*n* = 9) rats with preBötC lesions. Data are represented as median with 70% range (TNFα, MCP-1, MIP, and RANTES), and as mean ± SD (IFNγ, IL-1a, IL-1b, IL-6, and CRP) with individual data points. Data are represented as mean ± SD, with individual data points.

Hypercapnic hypoxia induces chronic inflammation (Maniaci et al., 2021) in patients with SA (Mcnicholas, 2009). Therefore, we measured the levels of inflammatory markers from inferior vena cava blood samples. Our cytokine panel showed rats with SA displayed increased levels of IFNγ, IL-1b, and MIP (Table 2; Figure 5) with no change in plasma levels of IL-1a, IL-6, TNFα, MCP-1, and RANTES (Table 2; Figure 5). Thus, this model of SA produces changes in specific inflammatory markers.

**Table 2:** Alterations of inflammatory markers in this model of sleep apnoea.

**Inflammatory** **marker**	**Sham**	**DTA**	**Significance**
*IFNγ*	2 ± 0.3, *n* = 11	2.8 ± 0.7, *n* = 9	*p* = 0.003
*IL-1b*	0.6 ± 0.1, *n* = 12	0.8 ± 0.1, *n* = 8	*p* = 0.049
*MIP*	14 R_70_ 1, *n* = 7	336 R_70_ 170, *n* = 9	*P* = 0.001
*IL-1a*	1.9 ± 0.1, *n* = 12	1.9 ± 0.1, *n* = 9	*p* = 0.96
*IL-6*	1.2 ± 0.0, *n* = 12	1.2 ± 0.0, *n* = 8	*p* = 0.6
*TNF-α*	16 R_70_ 9.8, *n* = 13	16 R_70_ 8.2, *n* = 9	*p* = 0.5
*MCP-1*	4.0 R_70_ 0.1, *n* = 12	4.0 R_70_ 0.1, *n* = 9	*p* = 0.9
*Rantes*	*2.1 R_70_ = 0.1, n = 10*	*2.3 R_70_ 0.5, n = 9*	*p = 0.3*

### Rats With Sleep Apnoea Have Neuroinflammation

**Figure 6:**
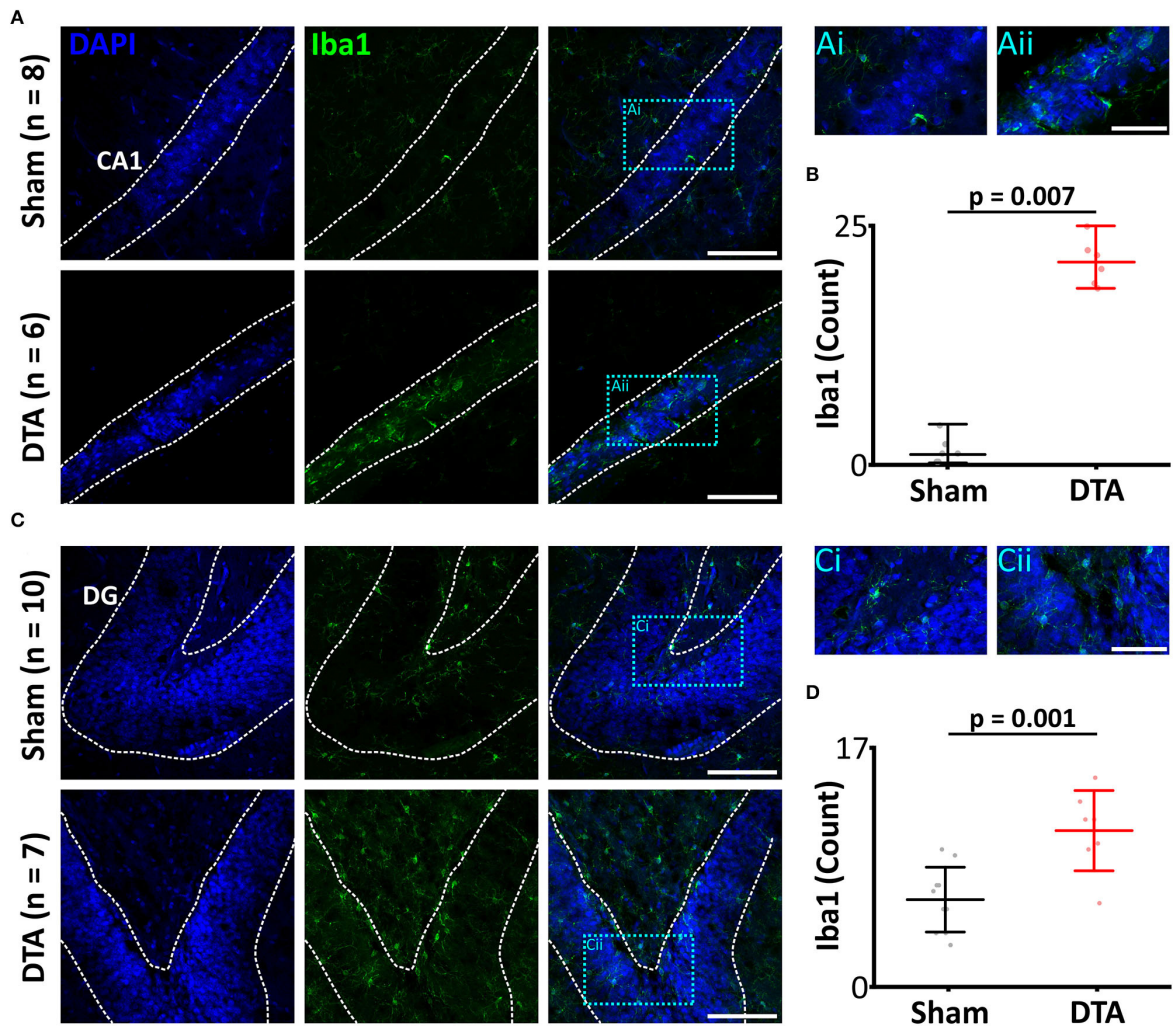
Induction of sleep apnoea leads to neural inflammation. **(A,C)** Micrographs showing activated microglia, a marker of neural inflammation, through Iba1 expression. Scale bars 100 μm. Blue boxes represent expanded areas shown in i and ii. Expanded micrographs scale bars 50 μm. **(A)** CA1 region and **(C)** Dentate gyrus (DG). **(B,D)** Group data are displayed in **(B)** CA1 and **(D)** DG. Data are represented as mean ± SD, with individual data points.

As IL-1b was elevated in SA rats and IL-1b is intimately linked to microglial activation (Monif et al., 2016) and neuroinflammation (Basu et al., 2004), we investigated Iba1 (a marker of activated microglia; Ito et al., 1998) levels in the hippocampus. SA rats displayed neuroinflammation in the hippocampus, which had elevated levels of activated microglia in the CA1 region (sham: 3 R_70_ 20 cells, *n* = 21 vs. DTA: 21 R_70_ 4 cells, *n* = 7: *p* = 0.007; Figures 6A,B) and dentate gyrus (sham: 5 ± 3 cells, *n* = 10 vs. DTA: 11 ± 3 cells, *n* = 7: *p* = 0.001; Figures 6C,D).

### Rats With Sleep Apnoea Have Neurophysiological Changes Resulting in Reduced LTP

**Figure 7:**
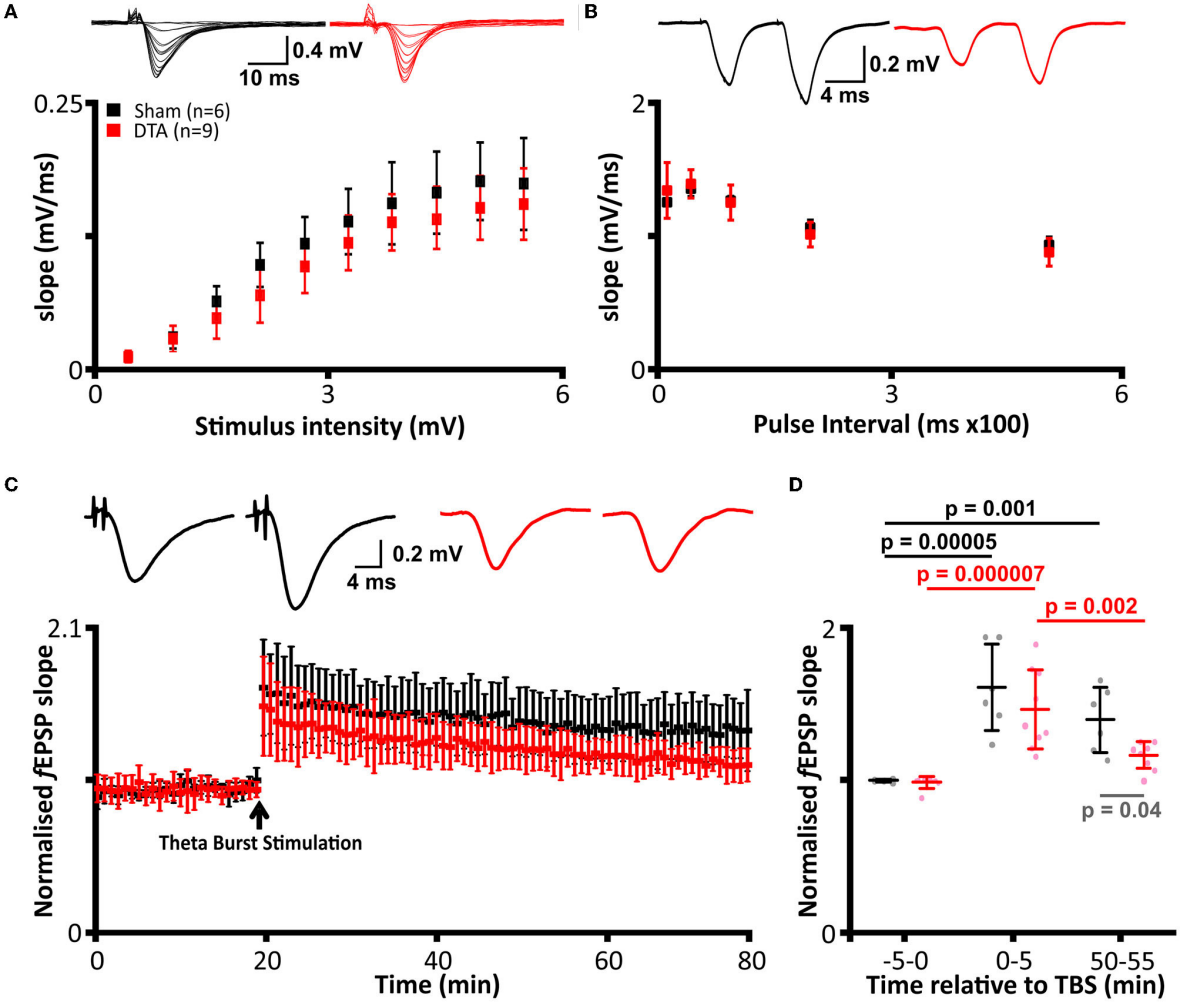
Induction of sleep apnoea has no effect on basal transmission but leads to loss of LTP. **(A–D)** DTA transduced rats (red) compared to shams (black). **(A)** Stimulus input-out curves. (Top) Representative data from individual rats and (bottom) group data. **(B)** Paired pulse facilitation. (Top) Representative data from individual rats and (bottom) group data. **(C)** Average recordings of the slope of field excitatory postsynaptic potentials (*f* EPSPs) from electrophysiological recordings before and after theta burst stimulation (TBS). (Top) Representative data from individual rats and (bottom) group data. **(D)** Group data from LTP traces represented in **(C)**. Analysis was performed on 5 min epochs immediately before and after TBS and following 1 h post-stimulation. Data are represented as mean ± SD.

We next tested to see if neuroinflammation in SA rats altered the electrophysiological properties in the hippocampus. We first investigated basal synaptic transmission in the CA1 region of the hippocampus. There was no difference in stimulus input-output curves between SA and sham rats (mean difference −18.9 ± 12.2 mV·ms^−1^; Figure 7A) and in the paired pulse ratio (mean difference 0.0009 ± 0.04; Figure 7B). Thus, basal synaptic transmission appears unaffected by sleep apnoea. To investigate long term potentiation (LTP), we used theta burst stimulation (TBS, see methods). SA rats showed decreased LTP following TBS. Both shams (mean difference −0.7; standard error 0.1, DF = 22; *p* = 0.000005) and SA rats (mean difference −0.5; standard error 0.1, DF = 22; *p* = 0.000007) showed significant short-term potentiation immediately following TBS (Figures 7C,D). However, short-term potentiation was not converted into long-term potentiation in SA rats, as the change in slope was reduced after 1 h (mean difference 0.3; standard error 0.1, DF = 22; *p* = 0.002) and was no longer different to baseline (mean difference −0.2; standard error 0.1, DF = 22; Figures 7C,D).

In contrast, sham rats displayed LTP, with their change in *f* EPSP remaining elevated at a level not different to that seen immediately after TBS (mean difference 0.2; standard error 0.1, DF = 22) and higher than baseline (mean difference −0.5; standard error 0.1, DF = 22; *p* = 0.001; Figures 7C,D). Overall, SA and sham rats had a similar baseline (mean difference 0.1; standard error 0.1, DF = 22) and STP (mean difference 0.2; standard error 0.1, DF = 22) *f* EPSP slopes but not LTP, which was diminished in SA rats (mean difference 0.3; standard error 0.1, DF = 22; *p* = 0.04). Thus, SA rats exhibited neurophysiological deficits, which could result in cognitive impairment.

### Rats With Sleep Apnoea Have Deficits in Short-Term Memory

**Figure 8:**
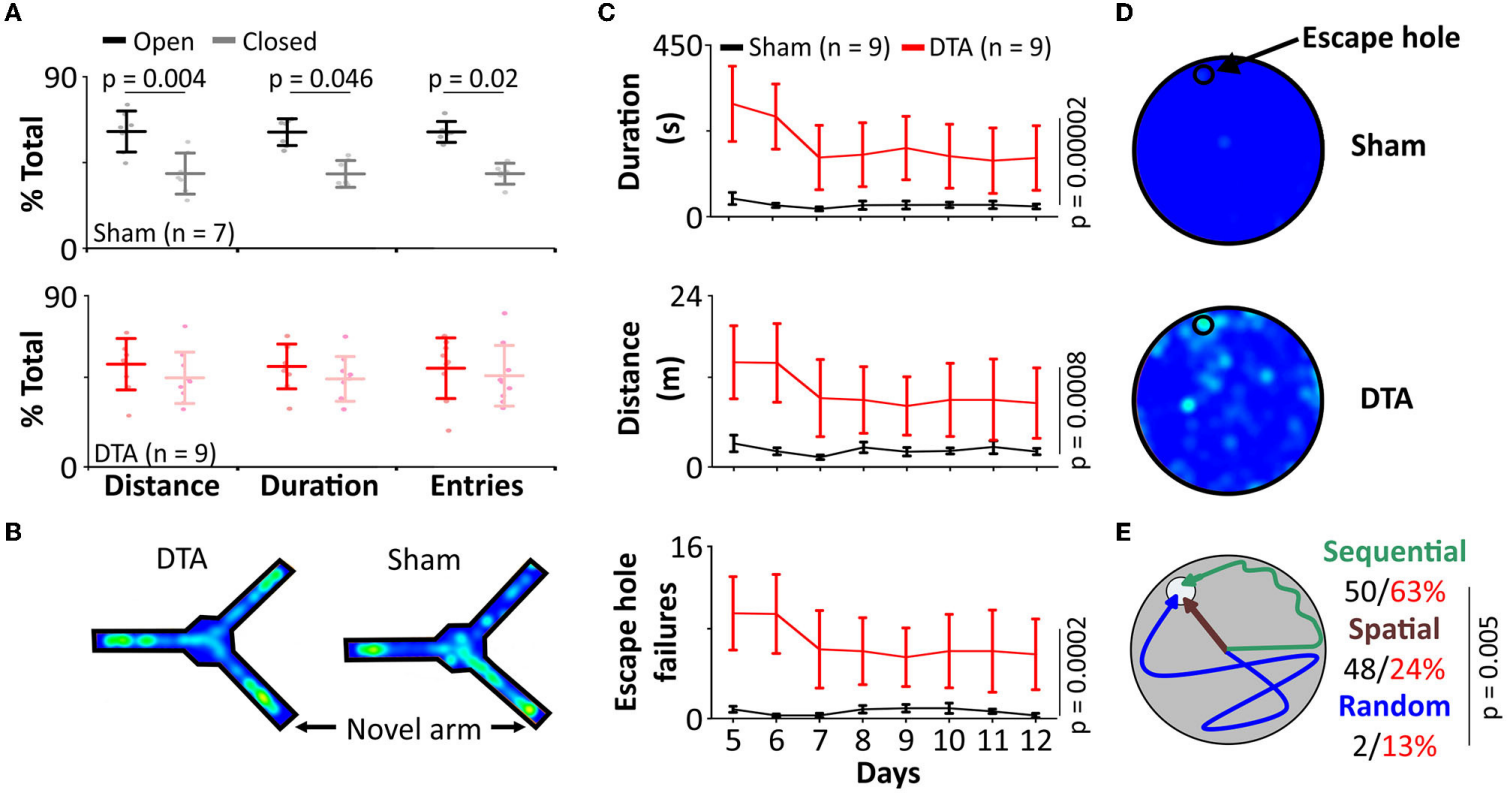
Our model of sleep apnoea exhibits cognitive decline. **(A,B)** Y-maze (forced alteration). **(A)** Group data showing number of entries, distance covered, and time spent in the novel arm (light shading) than the previously open arm (dark shading) in DTA transduced rats (red) compared to shams (black). **(B)** Heat maps from individual rats. **(C–E)** Barne's maze. **(C)** Group data showing time spent and distance covered on the maze, and the number of escape hole failures in DTA transduced rats (red) compared to shams (black). **(D)** Heat maps from individual rats. **(E)** Cartoon displays search strategies used by DTA transduced rats (red) compared to shams (black).

Neuroinflammation contributes to cognitive impairment by depletion of synapses and reduction in plasticity (Ma et al., 2014). We sought to assess how the neuroinflammation we observed in our SA rats affected learning and memory. In a Y-maze forced alteration test (short term memory; tests separated by 1 h), rats with SA displayed no preference for entries into (54% ± 4%; mean difference 7; standard error 5, DF = 14), duration in (52% ± 5%, mean difference 4; standard error 6, DF = 13) and distance traveled (53% ± 4%; mean difference 7; standard error 4, DF = 14) in the novel vs. open arm (Figures 8A,B). In contrast, shams displayed a strong preference for the novel arm (entries: 61% ± 4%, mean difference 22; standard error 6, DF = 14; *p* = 0.02; duration: 57 ± 5%, mean difference 22; standard error 7, DF = 13; *p* = 0.046; distance traveled: 61 ± 3%, mean difference 22; standard error 5, DF = 14; *p* = 0.004; Figures 8A,B). Interestingly, the discrimination ratio (novel/all arms) showed a decrease in the number of entries (sham: 46% R_70_ 15%, *n* = 4 vs. DTA: 30% R_70_ 13%, *n* = 9; *p* = 0.03; data not shown) and distance traveled in the novel arm (sham: 44% R_70_ 11%, *n* = 5 vs. DTA: 35% R_70_ 17%, *n* = 9; *p* = 0.049; data not shown) but not the time spent in the novel arm (sham: 31% R_70_ 14%, *n* = 7 vs. DTA: 32% R_70_ 11%, *n* = 9; data not shown). The data collected on the Y-maze was not confounded by anxiety or locomotor issues, as rats covered the same distance (sham: 15 ± 5 m, *n* = 7 vs. DTA: 17 ± 5 m, *n* = 9; data not shown).

### Rats With Sleep Apnoea Have Deficits in Long-Term Memory

As SA rats displayed impaired short-term memory, we next investigated whether they also showed deficits in the conversion of short- to long-term memory. Rats with SA spent longer to find the exit hole in the Barnes maze in terms of duration (sham: 31 ± 25 s vs. DTA: 189 ± 22 s; mean difference 158; standard error 35; DF = 14; *p* = 0.00002) and distance covered (sham: 2.4 m ± 1.5 m vs. DTA: 10.6 ± 1.3 m; mean difference −8; standard error 2; DF = 14; *p* = 0.0008, Figures 8C,D). SA rats failed to use the escape hole more often (sham: 7 ± 1 vs. DTA: 4 ± 2, mean difference −10; standard error 2; DF = 14 *p* = 0.0002; Figures 8C,D). Furthermore, SA rats were less likely to utilize spatial cues (sham: 48 ± 17% of trials vs. DTA: 24 ± 4%; of trials *n* = 8 per group; *p* = 0.005) instead preferring a sequential search strategy (64% of trials), with 6 times as many SA rats utilizing a random search strategy (sham: 2% of trials vs. DTA: 13% of trials) (Figure 8E).

## Discussion

Recently, SA has been gathering attention. Its far-reaching effects as a comorbidity for many diseases makes it a promising target for understanding the mechanisms of those diseases as well as providing new therapeutic targets. The major issue is that until now, there have been no good models for studying SA.

We created a stable lesion of the inspiratory oscillator using a virus to transduce cells to produce the alpha subunit of diphtheria toxin A (DTA). If lesion size is restricted, then loss of respiratory rhythm generating neurones in the preBötC should be compensated for during wakefulness (Mckay and Feldman, 2008). However, during sleep, reduction in chemosensory drive and inhibition of afferent inputs to the respiratory system make breathing more fragile, leading to apnoeas (Mckay and Feldman, 2008). Transduced rats developed sleep disordered breathing that closely resembles the breathing patterns observed in patients with SA.

Whilst we did not record from respiratory muscles, it is likely these apnoeas are central in origin given that obstructive apnoeas are not seen with unilateral ablation of the preBötC (Mckay and Feldman, 2008). Furthermore, arousals in our model were not coincident with the termination of an apnoea, instead arousals were associated with post apnoeic hyperpnoea responsible for reoxygenation; this is indicative of CSA rather than OSA (Jordan et al., 2011; Simms et al., 2013). Nevertheless, as central and obstructive sleep apnoeas can neither be distinguished by oxygen desaturation (Holleboom et al., 2020) nor the sleep state in which they occur (Loadsman and Wilcox, 2000; Gupta et al., 2016), this model constitutes a good representation of both diseases in terms of all but the changes in thoracic pressure seen in OSA.

Furthermore, respiratory changes during wakefulness in rats with SA were similar to the clinical presentation where untreated patients with SA display increased *f* with reduction in V_T_, thus maintaining V_e_ (Zaremba et al., 2016). Changes in breathing are unlikely to be due to any changes in metabolism, as the preBötC does not project to any nuclei known to be involved in metabolism, with the exception of the lateral hypothalamus (LH) (Tan et al., 2010; Yang and Feldman, 2018). However, only ~50% of neurones in the LH are involved in glucose sensing (Oomura et al., 1969), projections from the preBötC are sparse (Yang and Feldman, 2018), and we only saw a loss of 25% of preBötC neurones, making it unlikely there would be any significant change in metabolism through change in connectivity between the preBötC and LH. Additionally, although respiratory disturbances in SA rats likely lead to changes in blood gases, hypoxia does not cause metabolic changes in middle-aged rats (Haouzi et al., 2009). Finally, minute ventilation is affected by altered metabolism (Huang et al., 1984), whilst there were changes in frequency and tidal volume in SA rats, minute ventilation remains unchanged.

In addition to the respiratory similarities we found, the sleep-wake disturbances in our model mimic the disease. SA patients with and without CPAP showed no change in NREM sleep, but a 26% reduction in REM sleep occurs in patients with untreated SA, with all lost REM time being spent awake (numbers calculated from Table 1 in Aldrich et al., 1989); the same trend we observed in our rats. Alterations in REM sleep are directly linked to cognitive decline (Moe et al., 1995), and REM sleep disturbances are now thought to be an extremely reliable biomarker for Alzheimer's Disease (AD) (Holth et al., 2017). This sleep deprivation will contribute to a decrease in cognition as REM is crucial for memory consolidation (Boyce et al., 2016), and white matter integrity in older adults (Altendahl et al., 2020). Mechanisms by which REM sleep deprivation causes disruption of learning and memory and changes in the neurophysiology of the hippocampus can include perverse glucose metabolism (Kim et al., 2021), altered neurotransmitter levels (Saygin et al., 2017), inflammation (Yehuda et al., 2009), neuroinflammation (Ashley et al., 2016), and neural damage (Suresh et al., 2021).

**Figure 9:**
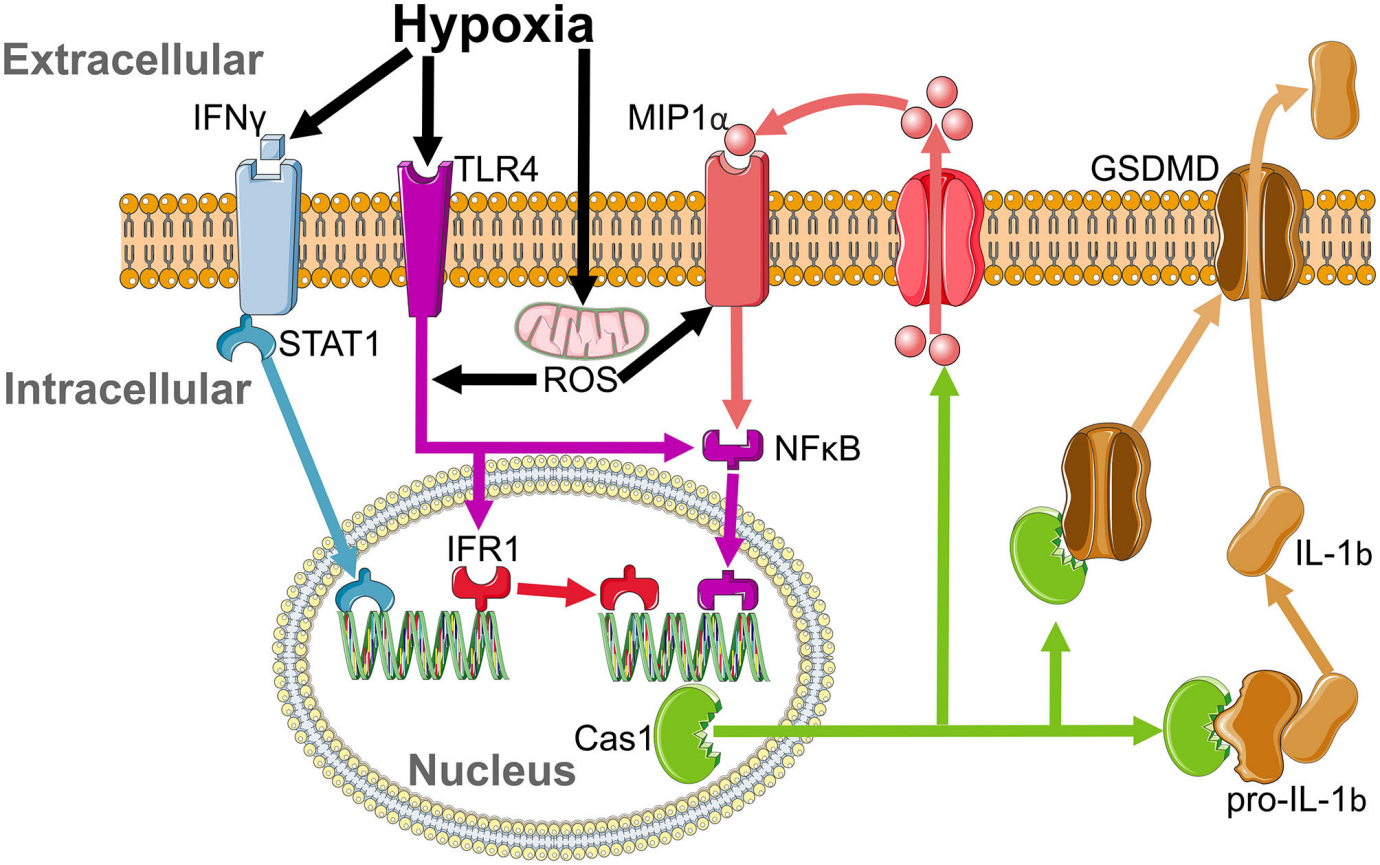
Proposed inflammatory cascade for sterile inflammation in sleep apnoea. IFNγ is elevated by hypoxia and activates interferon regulatory factor 1 (IRF1) by stimulation of STAT1. IFR1 acts as a transcription factor that drives the production of caspase 1. Activation of the MIP1αR and toll like receptor 4 (TLR4) pathways by reactive oxygen species (ROS) released from mitochondria during hypoxia enhances caspase 1 production by activation of NFKB. Caspase 1 cleaves pro-IL-1b to release its active form, IL-1b, which leads to the insertion of GSDMD, which allows for the translocation of IL-1b from the intracellular to extracellular space and increases MIP1a release.

Remarkably, we found only a select panel of inflammatory markers elevated in the plasma of our moderate SA model; IFNγ, IL-1b, and MIP. IFNγ activates interferon regulatory factor 1 (IRF1) (Ning et al., 2010) by stimulation of STAT1 (Castro et al., 2018) (Figure 9), with an increase in IRF1 following IFNγ-induced M1 polarization of macrophages (Martinez et al., 2006). IFR1 drives transcription of caspase 1, which cleaves pro-IL-1b to produce the active IL-1b protein (Tsuchiya et al., 2021) (Figure 9). Therefore, modulation of IL-1b is via post-transcriptional modification rather than increased transcription; hence IL-1b protein is increased even though paradoxically its mRNA is diminished. Caspase 1 further aids IL-1b release by cleaving Gasdermin D (GSDMD) to form the pore responsible for IL-1b release (Tsuchiya et al., 2021) (Figure 9). Once released, IL-1b induces secretion of MIP2 and MIP1β from parenchymal cells (Rock et al., 2010). In parallel, IFNγ induces both MIP1α and MIP1β release from macrophages (Martin and Dorf, 1991), likely in a caspase 1-dependent manner (Liang et al., 2010). This leads us to propose this as a possible mechanism by which moderate SA may affect neural inflammation (Figure 9), and by extension cognition.

Interestingly, whilst IFNγ activation evokes IL-1b and MIP secretion, it cannot by itself induce release of IL-6 (Martin and Dorf, 1991), RANTES (Marfaing-Koka et al., 1995), or MCP-1 (Yamana et al., 2009). Given IL-6 is responsible for inducing CRP release from the liver (Castell et al., 1989), it is therefore not surprising that plasma levels of CRP also remain unchanged. In conjunction, whilst IFNγ-activated macrophages release TNFα (Vila-Del Sol et al., 2008), this is inhibited by hypercapnia (Wang et al., 2010). Hence, TNFα would not be expected to be elevated in response to moderate SA. Finally, cleavage of pro-IL-1a to IL-1a occurs through a calpain-dependent, caspase-independent pathway (Tsuchiya et al., 2021). Calpain is inhibited by oxidative stress (Guttmann and Johnson, 1998), while caspase 1 is activated by it (Kim et al., 2003; Zhang et al., 2003), providing a basis for why IL-1a and IL-1b are differentially regulated during SA.

Hypoxia plays an important role in sterile inflammation. Lowered O_2_ increases plasma IFNγ (Roman et al., 2010) and induces toll like receptor 4 (TLR4) (Kim et al., 2010) and MIP1α receptor (Cowell et al., 2002) upregulation and activation. Increased IFNγ intiates IRF1-dependent inflammation, which is enhanced by hypoxia-induced ROS stimulation of the TLR4 pathway (Dhupar et al., 2011). Furthermore, upregulation of MIP (Kim et al., 2017) and TLR4 (Laghlali et al., 2020) will enhance caspase 1 production by NFκB activation, amplifying cleavage of pro-IL-1b to its functional form (Kim et al., 2003) and providing a second possible pathway by which hypoxia may lead to neural inflammation (Figure 9). Hence, IFNγ, IRF1, Caspase 1, TLR4, and MIP all drive microgliosis (Kim et al., 2003; Abd-El-Basset et al., 2020). Co-activation of TLR4 and the IFNγ receptor causes significant neuronal dysfunction and cell death (Papageorgiou et al., 2016), with neuronal cell death increased by hypoxia-induced caspase activity (Zhang et al., 2003). Therefore, hypoxia leads to IFNγ-induced microglial activation, which stimulates IRF1 to promote Caspase 1 production to cleave pro-IL-1b, a pathway enhanced by activation of TLR4 and MIP1α by ROS, leading to microgliosis and neuronal damage (Figure 9). These effects on neural inflammation may be why IFNγ is inversely correlated with cognitive ability (Monteiro et al., 2016) and grey matter volume in the dorsal hippocampus (Monteiro et al., 2016), and may therefore contribute to grey matter loss and mild cognitive impairment (MCI) in patients with SA (Torelli et al., 2011).

Microglial activation induces tau phosphorylation (Yoshiyama et al., 2007) in neurones and promotes conversion of MCI to both Alzheimer's Disease (AD) and Vascular Dementia (VaD) (Olsson et al., 2013). In addition to tau phosphorylation, SA contributes to AD through greater amyloid burden (Bu et al., 2015). SA is independently associated with increased dementia and MCI such as reduced memory or executive function (Sharafkhaneh et al., 2005; Pan and Kastin, 2014), with executive function being more vulnerable than memory, or core intellectual and verbal abilities (Aloia et al., 2004; Saunamäki and Jehkonen, 2007). Accordingly, treatment of SA is able to partially reverse this cognitive decline (Borak et al., 1996; Ooms and Ju, 2016). The loss of REM sleep, the activation of microglia, and the direct effects of inflammation on hippocampal size and function, may be why SA has such profound effects on the brain, and explains the deficits of long and short-term memory we saw in our rats.

Our SA rats display elevated plasma noradrenaline levels, as do patients with SA (Ziegler et al., 1997). Increased catecholamines in plasma are indicative of hypertension, as such patients with SA are at increased risk of dying from cardiovascular events, e.g., heart failure (Gottlieb et al., 2010) and stroke (Redline et al., 2010), which may have an atherosclerotic component (Lui and Ip, 2012) due to systemic inflammation (Libby, 2006; Bosc et al., 2010). Patients with heart failure (HF) treated for OSA have reduced mortality (Javaheri et al., 2011) and morbidity (Lavergne et al., 2015), and every level of HF mortality is directly related to the severity of SA (Lanfranchi et al., 1999; Lévy et al., 2013). Therefore, this model of SA may also be useful in studying the mechanism linking SA with cardiovascular comorbidities, including VaD, which is elevated in SA patients at higher rates than AD. The severity of cognitive decline in patients with VaD is positively correlated with SA severity (Culebras and Anwar, 2018), with treatment of OSA improving clinical outcomes (Culebras and Anwar, 2018).

In summary, we have developed an improved model of moderate SA that exhibits all of the clinical presentations tested. This model will be a valuable new research tool for investigation of SA directly and as a comorbidity for other disorders such as neurodegeneration.

## Data Availability Statement

The authors confirm the data supporting the findings of this study are available in the article, and are available from the corresponding author upon reasonable request.

## Ethics Statement

All experiments involving animals were approved by the University of Warwick Animal Welfare and Ethical Review Board in accordance with the United Kingdom Animals (Scientific Procedures) Act (1986) and the EU Directive 2010/63/EU, and performed under a project license issued by the United Kingdom Home Office.

## Author Contributions

RR performed experiments and analyzed data from all aspects of the project. MW performed and designed LTP experiments, helped design the Barnes maze experiments, and helped in writing the manuscript. IB designed and made the virus. KD performed plethysmography and ELISAs and analyzed ELISA data. AE performed plethysmography and ELISAs. JD performed plethysmography and Barne's maze and Y-maze experiments. SN performed immunocytochemistry. RH designed and performed experiments, analyzed data from all aspects of the project, and oversaw the project. All authors commented on the manuscript. All authors contributed to the article and approved the submitted version.

## Funding

This work was supported by an MRC Discovery Award MC_PC_15070 and Medical and Life Sciences Research Fund bursary.

## Conflict of Interest

The authors declare that the research was conducted in the absence of any commercial or financial relationships that could be construed as a potential conflict of interest.

## Publisher's Note

All claims expressed in this article are solely those of the authors and do not necessarily represent those of their affiliated organizations, or those of the publisher, the editors and the reviewers. Any product that may be evaluated in this article, or claim that may be made by its manufacturer, is not guaranteed or endorsed by the publisher.
